# Chimeric β-Lactamases: Global Conservation of Parental Function and Fast Time-Scale Dynamics with Increased Slow Motions

**DOI:** 10.1371/journal.pone.0052283

**Published:** 2012-12-21

**Authors:** Christopher M. Clouthier, Sébastien Morin, Sophie M. C. Gobeil, Nicolas Doucet, Jonathan Blanchet, Elisabeth Nguyen, Stéphane M. Gagné, Joelle N. Pelletier

**Affiliations:** 1 PROTEO, the Québec Network for Research on Protein Structure, Function and Engineering, Université Laval, Laval, Québec, Canada; 2 Département de Chimie, Université de Montréal, Montréal, Québec, Canada; 3 Département de Biochimie, Microbiologie et Bioinformatique, Université Laval, Laval Québec, Canada; 4 INRS–Institut Armand-Frappier, Université du Québec, Laval, Québec, Canada; 5 Département de Biochimie, Université de Montréal, Montréal, Québec, Canada; Wake Forest University, United States of America

## Abstract

Enzyme engineering has been facilitated by recombination of close homologues, followed by functional screening. In one such effort, chimeras of two class-A β-lactamases – TEM-1 and PSE-4 – were created according to structure-guided protein recombination and selected for their capacity to promote bacterial proliferation in the presence of ampicillin (Voigt *et al*., Nat. Struct. Biol. 2002 9:553). To provide a more detailed assessment of the effects of protein recombination on the structure and function of the resulting chimeric enzymes, we characterized a series of functional TEM-1/PSE-4 chimeras possessing between 17 and 92 substitutions relative to TEM-1 β-lactamase. Circular dichroism and thermal scanning fluorimetry revealed that the chimeras were generally well folded. Despite harbouring important sequence variation relative to either of the two ‘parental’ β-lactamases, the chimeric β-lactamases displayed substrate recognition spectra and reactivity similar to their most closely-related parent. To gain further insight into the changes induced by chimerization, the chimera with 17 substitutions was investigated by NMR spin relaxation. While high order was conserved on the ps-ns timescale, a hallmark of class A β-lactamases, evidence of additional slow motions on the µs-ms timescale was extracted from model-free calculations. This is consistent with the greater number of resonances that could not be assigned in this chimera relative to the parental β-lactamases, and is consistent with this well-folded and functional chimeric β-lactamase displaying increased slow time-scale motions.

## Introduction

Over the last two decades, numerous recombination techniques have been applied to modify and improve various properties of proteins, including stability and substrate specificity. Recombination of naturally-evolved homologues may be less disruptive to protein folding than protein evolution by creation of random mutations, thus increasing the likelihood of identifying functional recombinants [1]. DNA shuffling and its variations have been the most commonly used techniques to recombine homologous genes toward the creation of libraries of chimeras [2]; functional selection or screening may then allow the identification of the desired improved properties. Because those methods are DNA-based, no structural information is included in the choice of recombination sites. In contrast to that approach, structure-guided protein recombination offers control of the recombination sites [3]–[6]. Prominent among these, the SCHEMA approach probes short-range residue interactions to predict the fragments of homologous proteins whose recombination is most likely to preserve structural integrity, by means of maximizing sequence variation while minimizing changes in inter-residue contacts. SCHEMA has been successfully applied to the recombination of proteins with varied folds, including cytochrome P450s [7], fungal cellulases [8] and β-lactamases [9], [10]. In one example, the application of SCHEMA to the creation of chimera libraries increased the proportion of well-folded proteins from an average of 42% to as high as 75%, while including a high number of substitutions per sequence [7].

β-lactamases are broadly-distributed bacterial enzymes. They hydrolyse the β-lactam ring of antibiotics such as penicillins and cephalosporins, rendering those compounds ineffective and thus resulting in widespread antibiotic resistance [11]. TEM-1 and PSE-4 are two class A β-lactamases that share 40% sequence identity as well as virtually superimposable structures, with RMSD = 0.98 Å (backbone atoms). The variety of β-lactam substrates each hydrolyzes, known as its substrate recognition spectrum, overlap yet hold distinguishing features such as the higher catalytic efficiency of PSE-4 for hydrolysis of carbenicillin. Those two ‘parental’ β-lactamases were previously recombined using SCHEMA to create libraries of β-lactamase chimeras, which were selected for fold and function [10], [12], [13].

Because each of these ‘parental’ proteins is highly characterized, the artificial chimeras produced from the structure-guided recombination of TEM-1 and PSE-4 β-lactamases allows a thorough comparative study of the effects of structure-guided chimerization on structure and function. Furthermore, a question that has attracted increasing importance over the past decade is the contribution of protein dynamics to facets of enzyme catalysis, including substrate binding and turnover [14]–[20]. Despite the relatively low sequence identity (40%) between these two class A β-lactamases, similarities in the molecular motions of TEM-1 and PSE-4 have been observed, consistent with their structural similarity [21]–[25]. Both were highly rigid, yet motions inferred based on the absence/broadened peaks in NMR spectra and on the basis of model-free calculations were more important in the vicinity of the active site, suggesting a potential relation to the catalytic mechanism shared by these highly homologous enzymes.

**Figure 1:**
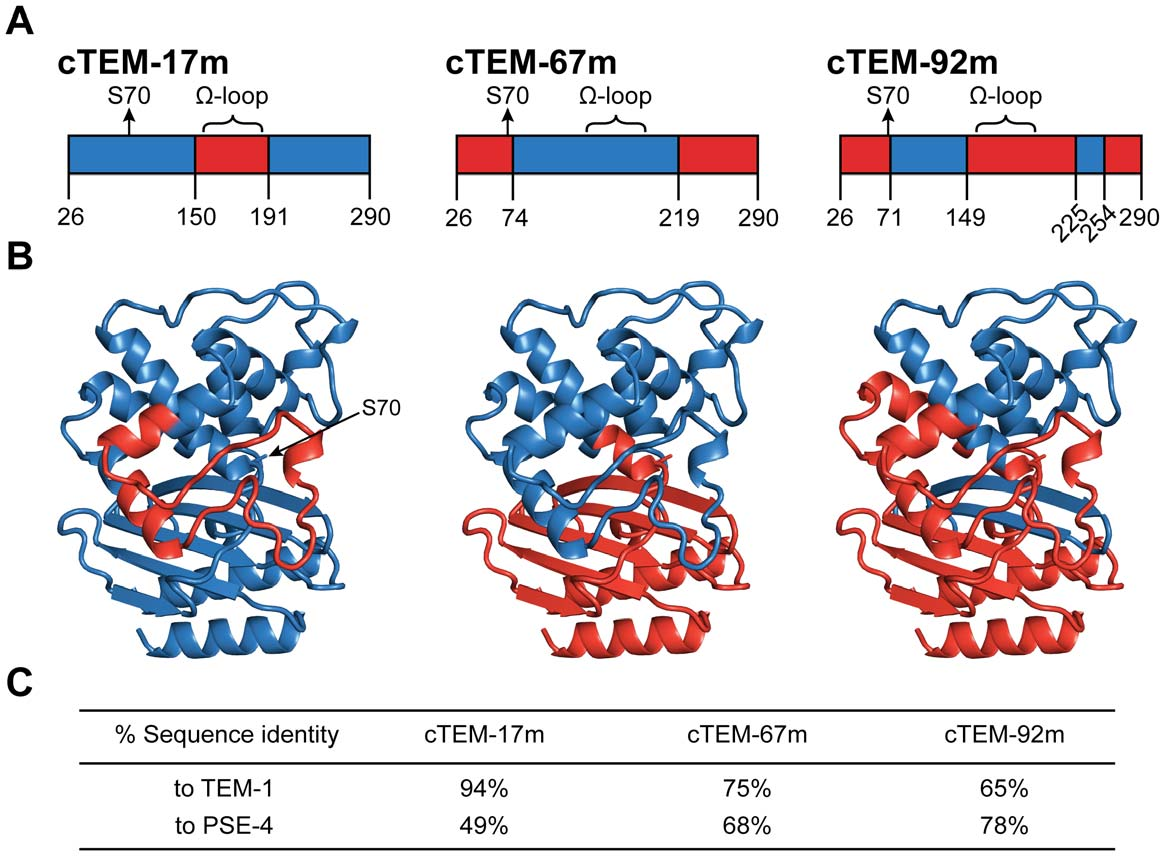
Sequence blocks exchanged between parental TEM-1 and PSE-4 β-lactamases in the selected, functional chimeras. A) Numbering of the sequence blocks originating from TEM-1 (blue) and PSE-4 (red) in cTEM-17m, cTEM-67m and cTEM-92m, according to the Ambler numbering [64]. The nomenclature of the chimeras is as follows: ‘c’ indicates chimeragenesis, and the number refers to the number of substitutions (or mutations: ‘m’) in each chimera relative to the TEM-1 parental sequence. The catalytic nucleophile, Ser70, and the catalytically-relevant Ω-loop, are indicated to highlight their parental origin. B) Structural representation of the sequence blocks exchanged during chimeragenesis, coloured according to parental origin as in panel A. The TEM-1 coordinates (PDB 1ZG4) were used for the representation. C) The sequence identity to each parental sequence is given. TEM-1 and PSE-4 differ by 150 residues (for a sequence identity of 40%) between residues 26 (the *N*-terminus following cleavage of the leader sequence of TEM-1) to 290 (*C*-terminus of TEM-1). The deletion at position 58 in PSE-4 is considered as a mutation relative to TEM-1. Thus, the additional four residues at the *N*-terminus and five residues at the *C*-terminus of PSE-4 are not included in the comparison to TEM-1.

The chimera cTEM-17m results from the structure-guided recombination of segments 26–149 (including the catalytic Ser70) and 191–290 from the TEM-1 parental β-lactamase, with segment 150–190 (encompassing the conserved Ω loop) from PSE-4 (Figure 1). In a previous study, we had ascertained the activity of the chimera cTEM-17m toward the synthetic, chromogenic substrate CENTA [26], indicating that the hydrolytic function of this chimera is not limited to hydrolysis of ampicillin, against which it had been selected [9]. We also assigned the backbone chemical shifts of cTEM-17m [26], confirming the highly parental-like fold of this chimera. These preliminary studies confirmed that the selected chimera cTEM-17m retained at least the most general structural and functional features of the parental sequences from which it was derived.

To gain insight into the tolerance of β-lactamases to inclusion of larger numbers of mutations derived from homologues, and thus gain a better understanding of laboratory evolution of enzyme by blockwise shuffling between homologues, we now extend our study to include more heavily substituted chimeras. Three TEM-1/PSE-4 chimeric β-lactamases are characterized in relation to the two ‘parental’ β-lactamases, to verify how a spectrum of blockwise sequence exchanges translates into similarities or differences from the ‘parental’ fold and functions. The three β-lactamase chimeras differ in the extent and in the identity of the sequence blocks that were swapped between the two parental sequences (Figure 1): one chimera is a closer homologue of TEM-1 (cTEM-17m), one is a closer homologue of PSE-4 (cTEM-92m) and one presents a balanced blend of both parental sequences (cTEM-67m). A distinguishing feature of any β-lactamase is their substrate recognition spectrum. As the substrate recognition spectra of TEM-1 and PSE-4 differ significantly, we assessed the effect of chimeragenesis on substrate specificity by kinetic analysis of the hydrolysis of β-lactam substrates belonging to various classes and generations.

We assessed the global structural features of the chimeric β-lactamases by performing CD melting curves. To gain more detailed insight into the effects of structure-guided protein recombination, we probed the protein backbone dynamics of the chimera cTEM-17m using NMR spin relaxation experiments. Chimera cTEM-17m was selected for this purpose because its chemical shift assignment has demonstrated that it is well behaved in solution and has a native-like fold [26]. As spin relaxation experiments were previously performed for the native TEM-1 and PSE-4 [23]–[25], this allowed order parameters (ps-ns timescale) and conformational exchange parameters (µs-ms timescale) from the parental TEM-1 and PSE-4 enzymes to be compared to those of this chimera containing 17 substitutions relative to TEM-1.

This study will further probe the strict conservation of various structural elements in class A β-lactamases. Moreover, it will shed additional light on the importance of conserved motions within a family of enzymes sharing the same structural scaffold, but differing in their sequence identity.

## Materials and Methods

### Materials

Oligonucleotide primers used for mutagenesis were synthesized by Integrated DNA Technologies (Coralville, IA) and by AlphaDNA (Montréal, QC). Kanamycin and isopropyl-1-thio-β-D-galactopyranoside (IPTG) were from Bioshop Canada (Burlington, ON). CENTA was purchased from Sigma-Aldrich (Oakville, ON) or synthesized according to the method of Bebrone *et al*. [27] with a 66% yield; ^1^H-NMR and positive mode ESI-MS showed only minor traces (<0.5%) of the cephalothin starting material. All other penicillin and cephalosporin substrates were obtained from Sigma-Aldrich.

### Chimera Subcloning into pET-24 with the OmpA Signal Sequence

The β-lactamase chimeras were subcloned out of the proTET or pMon vectors [10], [13], into the pET-24(+)a vector (kan^r^), where they were ligated 3' to the OmpA signal sequence for efficient expression and periplasmic export [28]. Chimera cTEM-17m, possessing the *N*- and *C*-termini of the TEM-1 parental sequence, was PCR-amplified using the terminal primers NdeIOmpATEMF and TEMHinDIIIR as previously described [29]. Chimeras cTEM-67m and cTEM-92m, possessing the *N*- and *C*-termini of the PSE-4 parental sequence, were PCR-amplified using the terminal primers NdeIOmpAPSEF (5'-CACACACACATATGAAAAAGACAGCTATCGCGATTGCAGTGGCACTGGCTGGTTTCGCTACCGTAGCGCAGGCCAGTAGTTCAAAGTTTCAGCAAGTT-3') and HinDIIIPSER according to the previously described method [29]. The primers NdeIOmpATEMF and NdeIOmpAPSEF contain the *Nde*I restriction site and the OmpA leader sequence. The resulting amplicons were sequentially digested with *Nde*I and *Hind*III (cTEM-17m) or *Pst*I (cTEM-67m, cTEM-92m) and ligated into pET-TEM-1 digested with the same enzymes and shrimp alkaline phosphatase-treated, before electroporation into *E. coli* BL21(DE3). Colonies were picked after selection on Luria-Bertani (LB) medium containing 30 µg/ml kanamycin. Because the chimeras had originally been produced using the *bla* gene from a commercial vector, the TEM-1 segments contained the Val84Ile and Ala184Val mutations, which differ from the native TEM-1 sequence. The chimeras were thus reverted to the native sequence by rolling-circle PCR using Pfx DNA polymerase (Invitrogen, Burlington, ON), mutagenic primers TEM-1 I84V (forward) 5′-GGCGCGGTATTATCCCGTGTTGACGCCGGG-3' and (reverse) 5′-CCCGGCGTCAACACGGGATAATACCGCGCC-3' or primers.

TEM-1 V184A (forward) 5′-GACACCACGATGCCTGCAGCAATGGCAACAACG-3' and (reverse) 5′-CGTTGTTGCCATTGCTGCAGGCATCGTGGTGTC-3'. Following *Dpn*I digestion (New England Biolabs, Ipswich, MA), the DNA was transformed as above. The entire sequence of each chimera was confirmed by DNA sequencing.

### Protein Expression and Purification

^15^N-labeled protein overexpression and purification for NMR data acquisition was achieved using previously published protocols [26]. Briefly, overexpression was performed in M9 minimal medium made with ^15^NH_4_Cl for ∼20 h with 0.4 mM IPTG at 25°C. Pelleted cells were lysed by one passage through a cell disrupter (Constant Systems) adjusted to 27 kpsi. The purification involved an anion exchange step (HiPrep 16/10 QXL, GE Healthcare) followed by gel filtration (HiLoad 26/60 Superdex 75 prep grade, GE Healthcare). Proteins used for enzyme kinetics and circular dichroism (CD) characterization were also expressed and purified according to previously established protocols [30], [XPATH ERROR: unknown variable "next".]. Briefly, overexpression was performed in Luria-Bertani broth. Cells were propagated at 37°C until OD_600_ = 0.6–0.8 followed by induction with 1 mM IPTG at 28°C for 16 h. Pelleted cells were lysed by one passage through a cell disrupter (Constant Systems) adjusted to 27 kpsi and centrifuged. The supernatant was filtered through a 0.2 µm filter. Purification was performed using Fast-Flow DEAE-Sepharose (Sigma-Aldrich). Yields were typically 25 mg/L, 18 mg/L, and 7 mg/L for chimeras cTEM-17m, cTEM-67m, and cTEM-92m, respectively.

### Circular Dichroism (CD)

CD spectra were recorded on a Jasco J-815 spectropolarimeter with a Peltier element for temperature control. Protein solutions were prepared at a final concentration of 0.1 mg/mL (3.5 µM) in 50mM sodium phosphate buffer, pH 7.0. The path length of the quartz cell was 0.2cm. Spectra were acquired at 25°C over the range 200–260 nm at a scan rate of 50 nm/min. Three scans were averaged. Buffer background recorded under the same conditions was subtracted. Two independent protein preparations were tested for all proteins except for cTEM-67m (single preparation). Thermal denaturation experiments were performed by monitoring changes in ellipticity at 222 nm, at a scan rate of 20°C/hour. The Savitzky-Golay method was used for curve smoothing [32]. The T_m_ values were determined using the derivatives function of the Jasco Spectra Manager software using the Savitzky-Golay algorithm for determination of first order derivatives, where T_m_ is given as the maximum of this derivative.

### Thermal Scanning Fluorimetry Shift Assays

Determination of the T_m_ values was performed by thermally-induced incorporation of SYPRO Orange into the unfolding protein, with analysis using a Q-PCR thermal cycler as previously described [33]. Briefly, each combination of 5 ×, 3.33 × and 2.5 × SYPRO Orange solution (Invitrogen) with 4 µM, 2 µM or 1 µM test protein was probed in a 96-well LightCycler plate (Sarstedt). SYPRO Orange and the protein were diluted with 50 mM sodium phosphate, pH 7, to a final volume of 20 µL per well. Controls contained SYPRO Orange in buffer. The plates were sealed using Optically Clear Sealing Tape (Sarstedt) and heated from 20°C to 95°C in a LightCycler 480 apparatus (Roche) with a ramp speed of 0.04°C/sec and 10 acquisitions/°C. Fluorescence was monitored with a CCD camera, using λ_exc = _483nm and λ_em_ = 568 nm and a 1 s exposure time. Any curve showing a maximum fluorescence plateau during denaturation was excluded from the T_m_ calculation.

For the T_m_ calculations, both temperature and fluorescence data were smoothed [32]. The first derivatives dFluo or dTemp were calculated using the cubic spline interpolation. The preliminary maximum was determined to obtain the half-values left and right of it. The linear fit for the curve outside the half-values was calculated, followed by the calculation of the average deviation from the fit. If the maximum was below the detection limit (fit value + 3 × deviation), the T_m_ determination was considered uncertain. The quadric fit around the maximum was then calculated as follows to obtain T_m_. The first derivative of the quadric fit function (y-value) was set to 0 and the x-axis value (temperature) was resolved. Then, the average deviation of the curve points around the maximum from the quadric fit was calculated. If the relative deviation was greater than 5% the T_m_ values were rejected if the corresponding maximum was below the detection limit. However, T_m_ values with a maximum above the detection but a relative deviation greater than 5% were defined as uncertain.

### Steady-state Enzyme Kinetics

K_M_ and *k*_cat_ values for the hydrolysis of CENTA, benzylpenicillin (BZ), carbenicillin (CB), cephalotin (CF), cefazolin (CZ), and cefotaxime (CTX) were determined at room temperature (21°C) in 50 mM sodium phosphate buffer, pH 7.0. The following extinction coefficients (and concentration ranges) were used: Δε_405 nm = _6400 M^−1^cm^−1^ for CENTA [27] (35–1000 µM), Δε_232 nm_ = 900 M^−1^cm^−1^ for BZ [34] (5–240 µM), Δε_232 nm_ = 1190 M^−1^cm^−1^ for CB [22] (5–240 µM), Δε_262 nm_ = 7960 M^−1^cm^−1^ for CF [34] (10–220 µM), Δε_260 nm_ = 7900 M^−1^cm^−1^ for CZ [35] (10–240 µM), and Δε_264 nm_ = 7250 M^−1^cm^−1^ for CTX [36] (40–300 µM). Substrate hydrolysis was monitored according to initial velocity for six substrate concentrations generally flanking the K_M_ values for WT TEM-1 (where allowed by the extinction coefficients and the K_M_ values) using a Cary100 Bio UV-visible spectrophotometer (Agilent Technologies Canada Inc., Mississauga, ON) and quartz cuvettes with a path length of either 1 cm (CF, CZ, and CTX) or 10 cm (BZ and CB), or a DTX 880 plate-reader (Beckman Coulter, Brea, CA) and 96-well plates (Corning Costar flat-bottomed model 9017) with 200 µL giving a path length of 0.7 cm (CENTA). Enzyme concentrations were held at 20–40 nM. The kinetic parameters for CF, CZ, and CTX were determined from the rates of hydrolysis calculated from the initial linear portion of the curve and were fitted to a Lineweaver-Burk model (1/V *vs* 1/[S]) using Microsoft Excel due to being unable to saturate the enzyme, as commonly observed with these substrates. Fitting for CENTA, CB, BZ was to the Henri Michaelis-Menten equation using GraphPad Prism (San Diego, CA).

### NMR ^15^N Spin Relaxation

#### Data acquisition and processing

NMR samples for the cTEM-17m chimera were prepared from lyophilized protein previously dialyzed against H_2_O. Lyophilized protein was dissolved in a solution of 0.1% azide, 3 mM imidazole (a pH indicator) and 10% D_2_O at pH 6.7, to a final protein concentration of 0.4 mM [26]. NMR spin relaxation data was recorded at 31.5°C on either a Varian INOVA 500 MHz (Québec/Eastern Canada High Field NMR Facility, Montréal, Canada) or 600 MHz (Université Laval, Québec, Canada), each equipped with *z*-axis pulse field gradients triple resonance cold probes. ^15^N-R_1_ experiments were performed using a sensitivity-enhanced inversion-recovery pulse sequence developed by Kay and co-workers [37]. Relaxation delays of 10.9, 21.8 (×2), 43.6, 87.2, 174.4, 348.9 (×2), 697.7, 1395.4, and 1995.0 ms were used for the R_1_ experiments recorded at both fields. ^15^N-R_2_ experiments were performed using the BioPack pulse sequence from Agilent Technologies (Santa Clara, CA) [38]. Delay times of 10, 30 (×2), 50, 70, 90, 110 (×2), 130, 150, 170, and 190 ms were used at the two fields. {^1^H}-^15^N steady-state NOEs were obtained by acquiring spectra with and without ^1^H saturation applied before the start of the experiments using an established pulse sequence [37]. A saturation time of 4s was used for {^1^H}-^15^N-NOE experiments. To eliminate the potential effect of sample or field homogeneity degradation over time on measured exponential decays, relaxation delays were acquired in an interleaved manner [23], [29]. All NMR data were processed using NMRPipe/NMRDraw [39], peak amplitudes were measured in NMRView [40], and fits performed as previously described [24]. A consistency test of multiple field transverse relaxation data was performed within “relax” (version 1.2.14) [41], [42] as described earlier [24], [43].

#### Model-free analysis

The internal motional parameters were interpreted from the relaxation data according to the model-free formalism as implemented in “relax” (version 1.3.4) [41], [42], [44], [45]. Dual optimization of the model-free parameters and the global diffusion tensor was done according to d’Auvergne and Gooley [41], with modifications as in [24] (see below). Only residues for which data was available at the two magnetic fields (500 and 600 MHz) were analyzed. N-H bond vector orientations were extracted from a homology model for cTEM-17m (see below). The value used for the ^15^N chemical shift anisotropy was -172 ppm and the N-H bond length was set to 1.02 Å for comparison with data for both TEM-1 and PSE-4.

The global models for a local correlation time (τ_m_) parameter for each residue, and either sphere, prolate spheroid, oblate spheroid, and ellipsoid diffusion tensors were tested and optimized using residues contained within well-defined secondary structures (as found using DSSP [46]) and selected using Akaike information criterion (AIC) [47]. After convergence of the diffusion tensors, the one with the lowest AIC was selected and local model-free models were minimized for all residues using this diffusion tensor which was then fixed. The best model for each residue was then selected using the AICc (Akaike Information Criteria for small sample size) approach to data fitting, to prevent overfitting. Errors on the extracted local model-free parameters were obtained from 500 Monte Carlo simulations as previously described [24].

### Homology Modeling

Homology modeling and subsequent structural minimizations were performed using the MOE molecular modeling program, version 2009.10 (Chemical Computing Group, Montréal, QC), using a Precision 670 Dell Workstation running Windows XP. Energy minimizations, homology models, and protein geometry analysis were performed with the Compute module using the CHARMM22 force field and a distance dependent dielectric (ε = 1) as implemented in MOE.

Three-dimensional homology modeling for the cTEM-17m chimera was undertaken using a multiple template approach based on the crystallographic structures of TEM-1 and PSE-4 β-lactamases (TEM-1: PDB coordinates 1ZG4 [48], PSE-4: PDB coordinates 1G68 [22]). Sequence alignment was performed for cTEM-17m against the sequences of TEM-1 and PSE-4 using the MOE-Align module to determine the optimal regions of the template secondary structure to be used for model building. Twenty-five intermediate models were generated for cTEM-17m. The final model was constructed according to the Cartesian average of all intermediate models and was validated relative to steric and atomic clashes as well as side chain rotamer outliers to confirm model quality. Hydrogen atoms were added at the normal ionization state of amino acids at pH 7.0 and partial charges were fixed to the CHARMM22 atom types. The resulting structure was minimized using conjugate gradient minimization until a convergence of 0.001 kcal·mol^−1^·Å^−1^ was reached. During this procedure, potential steric clashes between residue side-chains were prevented by applying a tethering force constant of 1 kcal· mol^−1^·Å^−1^ to the backbone atoms of the protein.

## Results and Discussion

The generation of the chimeric β-lactamases was accomplished by Meyer *et al*. using the SCHEMA protein recombination algorithm [10], [13]. In the case of chimeras cTEM-17m and cTEM-67m, the cross-over points between sequence blocks were calculated to minimize disruption of intramolecular contacts upon recombination [10]. The recombination algorithm was then modified to include some cross-overs at sites of maximal disruption of intramolecular contacts; cTEM-92 results from cross-overs at two minima and two maxima [13]. From each pool of recombined TEM-1/PSE-4 β-lactamases, the functional chimeras were selected according to survival of *E. coli* in the presence of ampicillin. The functional chimeras were further qualified by determining minimum inhibitory concentrations (MICs) for ampicillin hydrolysis [9]. The chimeras’ MICs were in the same range as the native, parental β-lactamases, providing indirect evidence of the maintenance of a β-lactamase-like fold for these laboratory-evolved chimeras.

Naturally-occurring β-lactamases vary in their substrate recognition spectrum, and their recombination may further alter substrate recognition. To determine how chimera formation affected the fold and the substrate recognition spectrum of chimeras, we examined three chimeras differing broadly in their pattern of chimerization (Figure 1), while sharing a similar MIC for ampicillin hydrolysis [10], [13]. In these chimeras, elements of the active site are contributed from both parents. Indeed, the contribution of each parent to the active sites of chimeras cTEM-17m and cTEM-67m is essentially reversed, *i.e.* where an active site block is from TEM-1 in cTEM-17m, it originates from PSE-4 in cTEM67m. Chimera cTEM-92m presents a different pattern of active-site blending. By these means, the influence of different ‘blends’ of parental fragments on structure and function could be assessed. The interacting *N-* and *C*-terminal α-helices are from a single parent (whether TEM-1 or PSE-4) in these chimeras, as was predominantly observed in the functionally selected TEM-1/PSE-4 chimeras [9], [10], indicating a strong bias toward maintenance of the native helix-helix interaction for structure/function.

### Overall Fold and Stability of the Chimeras

**Figure 2:**
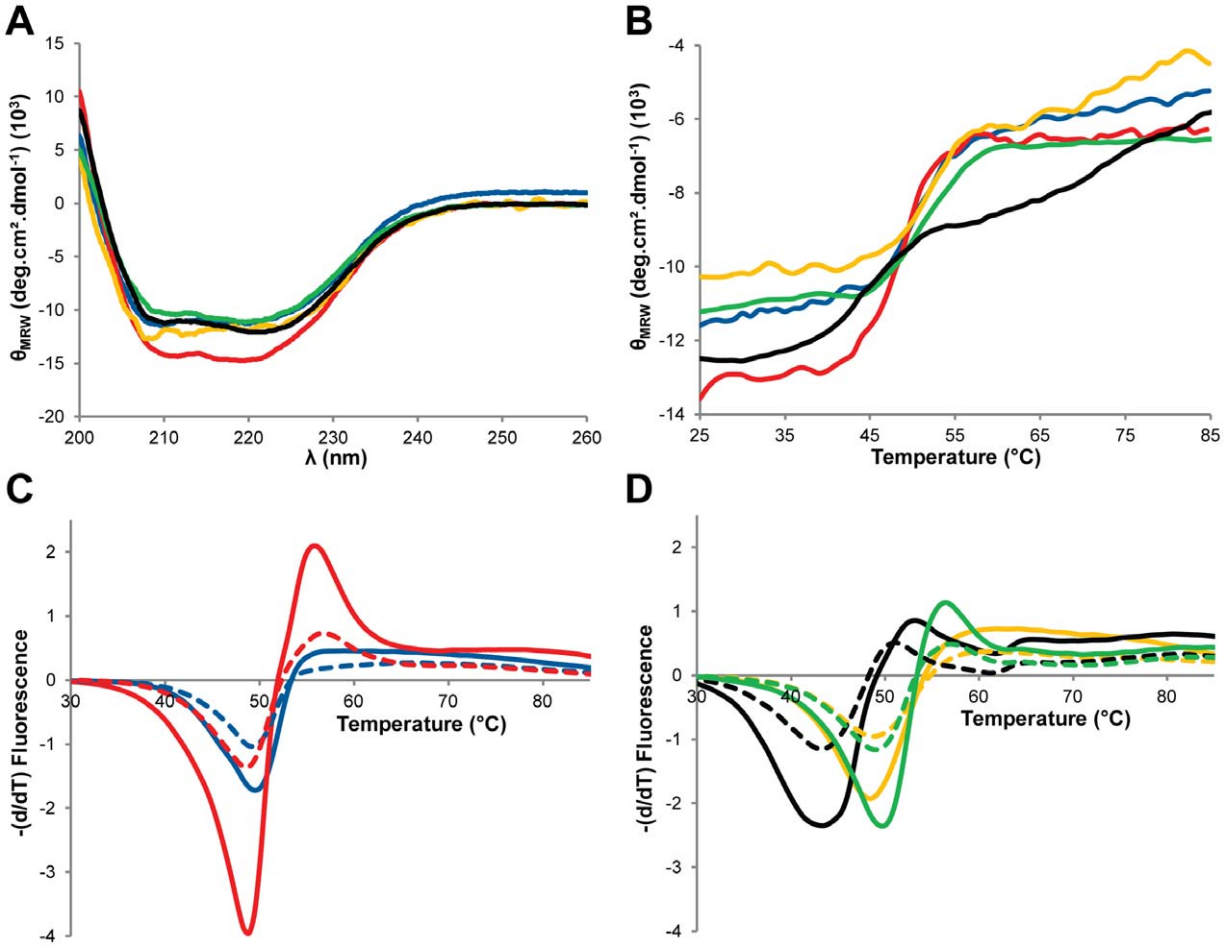
Thermal denaturation of parental and chimeric β-lactamases monitored by CD spectroscopy and thermal scanning fluorimetry. A) Far-UV CD spectra at 25°C of TEM-1 (blue), PSE-4 (red), cTEM-17m (gold), cTEM-67m (green) and cTEM-92m (black). B) Mean molar residual ellipticity (MRW) measured at 222 nm during thermal denaturation at a rate of 20°C/hour for TEM-1 (blue), PSE-4 (red) and the chimeras cTEM-17m (gold), cTEM-67m (green) and cTEM-92m (black). C, D) First derivative analysis of representative thermal melting curves observed by fluorescence of SYPRO Orange. Melting curves are shown for a ratio of 3.33 × SYPRO Orange to 1 µM (dashed line) or 2 µM (full line) protein, for C) TEM-1 (blue), PSE-4 (red) and D) the chimeras cTEM-17m (gold), cTEM-67m (green) and cTEM-92m (black).

To better assess the overall fold and stability of the chimeras, CD and thermal scanning fluorimetry shift measurements were performed. The far UV molar ellipticity spectra (200–260nm) of the parents are indistinguishable from each other, and from that of the three chimeras, consistent with the overall fold of the chimeras being similar to that of the parental β-lactamases (Figure 2A). To assess thermal stability of the chimeras, thermal denaturation was measured by CD at 222 nm (Figure 2B). The T_m_ determined for TEM-1 and PSE-4 were respectively 49.6°C and 48.6°C (Table 1). The T_m_ for TEM-1 compares well to a previously reported value (51.6°C) [49], while the T_m_ for PSE-4 is lower than a previous estimate (60°C) conducted by 10°C temperature jumps [50]. Thus, under our experimental conditions, the TEM-1 and PSE-4 homologues have similar T_m_ values.

**Table 1:** T_m_ values obtained upon thermal denaturation monitored by circular dichroism (CD) at 222nm and thermal scanning fluorimetry for TEM-1, the chimeric β-lactamases and PSE-4.

	CD	Fluorescence
	(°C)	(°C)
**TEM-1**	49.6	49.1±0.2
**cTEM-17m**	52.2	49.0±0.8
**cTEM-67m**	51.0	49.2±0.7
**cTEM-92m**	44.0	43.4±0.5
**PSE-4**	48.6	47.8±1.2

Using the thermal scanning fluorimetry shift approach, the thermal stability of the parents and the chimeras was verified. This method uses SYPRO Orange as a fluorescent dye for real time measurement of the protein fluorescence during denaturation [33]. SYPRO Orange has an affinity for hydrophobic regions of proteins, with a weaker fluorescence when free in solution. Figure 2C, D illustrates the first derivative of the thermal denaturation of the parental and chimeric β-lactamases. All T_m_ values are in agreement with those obtained by CD (Table 1), with cTEM-17m exhibiting the greatest inter-method variation (3.2°C). CD measures the denaturation of secondary structure elements while scanning fluorimetry monitors the exposure of the hydrophobic core of the protein during denaturation. The agreement of the values observed here indicates that denaturation of the main elements of secondary structure exposes the hydrophobic core. We note that PSE-4, cTEM-67m and cTEM-92m all show a pronounced maximum following the minimum representing the T_m_ (Figure 2C, D). This may be indicative of protein aggregation [51], where the two chimeras most similar to PSE-4 exhibit PSE-4-like properties, as opposed to cTEM-17m which is more TEM-1-like. Furthermore, we note that renaturation was not monitored.

The similar general aspect of the denaturation curves indicates that the fold of chimeras cTEM-17m and cTEM-67m is native-like despite the inclusion of numerous substitutions (17 and 67 substitutions relative to TEM-1, respectively). This is consistent with the native-like NMR chemical shift assignments obtained for cTEM-17m [26]. In contrast, the thermal denaturation of chimera cTEM-92m gave T_m_ values that were significantly lower than for all other parents and chimeras, being near 43–44°C relative to 48–52°C for all others. Its CD denaturation pattern shows a more gentle transition than the others, consistent with the first derivative minimum of scanning fluorescence denaturation that is broader than all others. These data indicate that the stability of cTEM-92m (92 substitutions relative to TEM-1; 58 relative to PSE-4) has been altered by recombination. Indeed, it may be considered surprising that cTEM-92m has conserved a native-like fold and activity (see below) at physiologically-relevant temperatures, given that its SCHEMA-calculated disruption score, or ‘*E*’, is significantly higher (*E* = 41) than *E* ≤26 that had previously been suggested as an upper limit of disruption still allowing function in β-lactamases [13]. A closer look at the blended segments (Figure 1B) reveals that only in cTEM-92m is the α/β domain constituted of segments from both parental enzymes. Thus, strand S4 (residues 244–251) is from TEM-1 and strand S5 (259–266) is from PSE-4, where three residues differ from TEM-1: residues 259, 262 and 265. In TEM-1, Arg259 forms a salt bridge with Glu48 [21], while in PSE-4 (and cTEM-92m) the salt bridge is lost as Ile259 packs against Val48 [22]. Nearby, Thr265 (TEM-1) is exchanged for Leu265 (PSE-4 and cTEM-92m). It has been suggested that the mutation of Thr265 to a methionine in TEM-1 would abolish the water molecule bridges involving Gly242 and Ser268, and hydrogen bonding with Arg244, thus contributing to structural modification in this region [21]. A similar effect on core packing may result from substitution of Thr265 with a leucine.

### Kinetic Properties of the Chimeras

While initial *in vivo* selection of the chimeras against ampicillin provided an indication of functional activity, a more rigorous catalytic characterization was required to fully assess the effects of structure-guided recombination on catalytic function. We previously reported that the hydrolytic activity of cTEM-17m, using the chromogenic cephalosporin substrate CENTA (Figure S1), is in the same range as the parental TEM-1 and PSE-4 β-lactamases [26]. Here, we determined that the more highly blended chimeras cTEM-67m and cTEM-92m were not as efficient in hydrolyzing this chromogenic substrate (Table 2).

**Table 2:** Kinetic constants for hydrolysis of the chromogenic substrate CENTA by TEM-1, the chimeric β-lactamases and PSE-4 a.

Substrate	Variant	K_M_	*k_cat_*	*k_cat_*/K_M_	*k_cat_*/K_M_ relative toTEM-1	*k_cat_*/K_M_ relative to PSE-4
		(µM)	(s^−1^)	(M^−1^s^−1^)		
**CENTA**	TEM-1	36±5	33±1	9.2 × 10^5^	1.00	2.2
	cTEM-17m	160±35	48±3	3.1 × 10^5^	0.34	0.74
	cTEM-67m	610±64	1.5±0.7	2.5 × 10^3^	0.003	0.01
	cTEM-92m	580±29	13±1	2.2 × 10^4^	0.02	0.05
	PSE-4	46±9	19±1	4.1 × 10^5^	0.45	1.00

a Values are given as the average +/− standard deviation from the mean.

To gain further insight into the substrate recognition spectrum and catalytic potential of the SCHEMA-based evolved chimeras, steady-state kinetic analyses were performed toward the following representative members of β-lactam substrates: two penicillins, benzylpenicillin (BZ) and carbenicillin (CB); two first-generation cephalosporins, cephalothin (CF) and cefazolin (CZ); and the third generation cephalosporin cefotaxime (CTX) (Figure S1). Despite the introduction of numerous substitutions as a result of structure-based recombination, the catalytic efficiency (*k_cat_*/K_M_) of cTEM-17m towards β-lactam hydrolysis is most similar to TEM-1 for all substrates tested (Table 3). Closer inspection revealed a 3 to 5-fold increase in K_M_ relative to TEM-1 for the cephalosporins CF and CZ. Interestingly, cTEM-17m was found to possess a slightly improved CTX recognition profile, with a 3-fold lower K_M_ for CTX *versus* TEM-1, though this was not selected for. The 17 mutations distinguishing TEM-1 and cTEM-17m are thus well tolerated with respect to function.

**Table 3:** Kinetic constants for the hydrolysis of penicillins and cephalosporins by TEM-1, the chimeric β-lactamases and PSE-4 a.

Substrate	Variant	K_M_	*k*_cat_	*k*_cat_/K_M_	*k*_cat_/K_M_ relativeto TEM-1	*k*_cat_/K_M_ relativeto PSE-4
		(µM)	(s^−1^)	(M^−1^s^−1^)		
**Benzylpenicillin (BZ)**	TEM-1	20±3	450±100	2.3 × 10^7^	1	0.6
	cTEM-17m	28±8	480±46	1.7 × 10^7^	0.7	0.4
	cTEM-67m	9.2±2	230±28	2.5 × 10^7^	1.1	0.6
	cTEM-92m	8.9±0.6	20±3	2.0 × 10^6^	0.1	0.1
	PSE-4	16±2	630±110	4.0 × 10^7^	1.7	1
**Carbenicillin (CB)**	TEM-1	49±12	92±4	1.9 × 10^6^	1	0.2
	cTEM-17m	22±4	37±2	1.7 × 10^6^	0.9	0.2
	cTEM-67m	54±13	62±15	1.1 × 10^6^	0.6	0.1
	cTEM-92m	64±8	500±18	7.8 × 10^6^	4.1	1
	PSE-4	64±5	500±92	7.8 × 10^6^	4.1	1
**Cephalothin (CF)**	TEM-1	180±28	84±12	4.7 × 10^5^	1	36
	cTEM-17m	470±80	120±8	2.4 × 10^5^	0.5	18
	cTEM-67m	58±26	0.005±0.001	9 × 10^1^	0.0002	0.007
	cTEM-92m	100±16	1.5±0.2	1.5 × 10^4^	0.03	1.2
	PSE-4	64±34	0.80±0.01	1.3 × 10^4^	0.03	1
**Cefazolin (CZ)**	TEM-1	130±9	55±8	4.2 × 10^5^	1	32
	cTEM-17m	600±90	75±21	1.2 × 10^5^	0.3	9.2
	cTEM-67m	51±12	0.004±0.001	8 × 10^1^	0.0002	0.006
	cTEM-92m	330±69	1.4±0.2	4.2 × 10^3^	0.01	0.3
	PSE-4	140±39	1.9±0.3	1.3 × 10^4^	0.03	1
**Cefotaxime (CTX)**	TEM-1	840±160	0.7±0.1	8 × 10^2^	1	5.5
	cTEM-17m	260±100	0.14±0.04	5.4 × 10^2^	0.7	3.6
	cTEM-67m	ND b	ND	ND	ND	ND
	cTEM-92m	220±97	0.3±0.1	1.4 × 10^3^	1.7	9.3
	PSE-4	200±30	0.03±0.01	1.5 × 10^2^	0.2	1

a Values are given as the average +/− standard deviation from the mean.

b ND: Not determined, the activity being too low or undetectable.

The structure-guided recombination of TEM-1 and PSE-4 to generate cTEM-67m involved blending the *N*- and *C*-terminal regions of PSE-4, (including the α/β-domain with the active-site residues 66–73) with the all-α domain of TEM-1 (including the catalytic Ω-loop residues 161 to 179) (Figure 1A, B). Despite the highly blended nature of cTEM-67m (67 substitutions relative to TEM-1; 83 relative to PSE-4), it readily hydrolyzed the penicillins BZ and CB with catalytic efficiencies in good agreement with the TEM-1 and PSE-4 parental enzymes (Table 3). However, catalytic efficiency for hydrolysis of the cephalosporins CF and CZ was reduced by 3 and 4 orders of magnitude relative to PSE-4 and TEM-1, respectively. Furthermore, cTEM-67m lost the capacity to hydrolyze CTX. Closer inspection reveals a 2–4 orders of magnitude reduction in *k_cat_* toward the cephalosporins, while K_M_ changed little or decreased.

These results indicate that even the apparently conservative substitutions resulting from structure-based recombination in cTEM-67m significantly modulate turnover of cephalosporin-based substrates. Previously, Huang *et al*. randomized the entire TEM-1 sequence (3 to 6 residues at a time) to probe sequence space plasticity [52]. That study found that 30 of the 263 positions tested in TEM-1 did not tolerate amino acid substitutions (in the context of concurrent point mutations) yet showed variation among naturally-occurring class A β-lactamases. Eleven among those 30 differ between TEM-1 and PSE-4: L30**E**, Y46**V**, **L**76A, **L**122A, **D**214N, **A**217T, K234**R**, G245**S**, L250**V**, G251**W** and W290**Y** (where L30**E** indicates Leu in TEM-1, Glu in PSE-4 and **Glu** in cTEM-67m while at position 76, it is the **Leu** originating from TEM-1 that is found in cTEM-67m). Among all the substitutions present in cTEM-67m, these 11 are likely to wield the most influence on catalytic function. It appears unlikely that substitutions to residues 30, 46, 250, 251 or 290 would affect kinetics in cTEM-67m because they are all at the structural core of the α/β domain which originates entirely from PSE-4 in this chimera, such that it is not disturbed. Additionally, neither Leu→Ala mutation (positions 76 and 122) is expected to have much functional impact as they are conservative and are within the core of the all-α domain, not facing the active-site cleft. The active site of cTEM-67m, however, where substitutions would have the greatest impact, lies at the interface of the α/β domain (originating from PSE-4) and the all-α domain (originating from TEM-1). Residues 214 and 234 coordinate the substrate carboxylate *via* a water molecule, and neighbouring residue 217 belongs to loop 213–220 that defines one of the ‘walls’ of the binding pocket. Their role in substrate binding is consistent with their substitution altering reactivity, though no structural insight on this alteration can be derived at this time. Finally, the G245S substitution is in the α/β domain, one strand removed from the active-site cleft and facing away from it; the proposed role of its conserved neighbour, Arg244, in substrate stabilization, may be perturbed.

The most structurally blended β-lactamase chimera considered in this study was cTEM-92m. It holds the most cross-over points between the two parental sequences, its four cross-overs occurring in the all-α domain, in the α/β domain, and in the corresponding inter-domain loop regions which connect the two domains together. It includes a similar number of substitutions from the parental sequences as cTEM-67m (92 substitutions relative to TEM-1; 58 relative to PSE-4); it is most similar to PSE-4 while cTEM-67m is closer to TEM-1 (Figure 1C). The cTEM-92m chimera showed the weakest catalytic efficiency for hydrolysis of BZ (decreased *k_cat_*). Nonetheless, it maintained a good efficiency for CB hydrolysis, mimicking its closest parent PSE-4 (Table 3). Hydrolysis of the first generation cephalosporins CF and CZ (but not the chromogenic CENTA) was also PSE-4-like with catalytic efficiencies 30–100% of the PSE-4 parent, and was distinct from TEM-1 due to a ≈ 50-fold reduction in *k_cat_*. Despite the highly mutated nature of cTEM-92m, this chimera had the highest catalytic efficiency for hydrolysis of the third generation cephalosporin CTX among the enzymes analyzed in this study, though CTX hydrolysis had not been selected for. This confirms that cTEM-92m maintains a well-folded structure at physiologically-relevant temperatures, and highlights the potential for structure-guided recombination to provide variants with functional diversity.

### Homology Model of the cTEM-17m Chimera

Using the TEM-1 (1ZG4 [48]) and PSE-4 (1G68 [22]) crystal structures, the residue 150–190 segment of PSE-4 was merged into the corresponding gap in the TEM-1 structure by homology modeling. The cTEM-17m homology model displayed a global backbone RMSD of 0.61 Å and 0.82 Å relative to the parental TEM-1 and PSE-4 structures, respectively, suggesting a general conservation of atomic packing in the chimera. In addition, analysis of the side-chain packing within the substrate binding pocket of the model showed that no significant backbone distortions were required to accommodate the 17 new residues from PSE-4 now included in the TEM-1 framework. This model was applied to subsequent analysis of NMR data.

### Dynamics of the cTEM-17m Chimera

As illustrated above, the three chimeras analyzed retained substrate recognition and turnover rates in the range of their most closely-related parent, with cTEM-67m being the most functionally crippled. This similarity to parental function – including a range of affinities toward β-lactam compounds belonging to different classes – suggests that those chimeras have a native-like fold. This has been confirmed by the native-like NMR spectra (and derived secondary structure) observed for the least mutated chimera, cTEM-17m [26] and by the CD results obtained in this study. A further property relevant to assessing the structure-function resemblance of a laboratory-evolved chimera to its parents is conservation of protein dynamics. A growing body of research [14]–[16], [18] indicates a substantial contribution of protein dynamics to enzyme function, such that the effect of structure-guided protein recombination on dynamics should be probed. To this effect, NMR backbone dynamic studies of the β-lactamase chimera cTEM-17m were performed. This chimera was chosen for study by NMR ^15^N spin relaxation experiments due to its structural and kinetic similarity to TEM-1, high expression levels (25 mg/L), solubility at the mM concentrations required for NMR studies and stability in solution over the 7-day span required for data acquisition (Figure S3). As NMR backbone dynamic studies of both parental β-lactamases have previously been performed [23]–[26], this study will enable determination of whether the chimera conserves the dynamic features of its parents. We note that it has not yet been possible to identify conditions where the chimeras cTEM-67m and cTEM-92m were sufficiently stable to allow similar NMR characterization.

The backbone chemical shift assignments for cTEM-17m were previously reported [26] (BMRB accession number 16598). Its well-folded β-lactamase-like structure was inferred from the well-dispersed nature of the H_N_ resonances, in very good agreement with the results obtained for the parental TEM-1 and PSE-4 (BMRB accession numbers 6024 and 6838, respectively [53], [54]). Confirmation of the conserved disulfide bridge (Cys77-Cys123) as well as the chemical shift-predicted secondary structure further established the well-folded nature of cTEM-17m. Despite yielding high-quality spectra, the 98% assignment completeness (^1^H_N_ and ^15^N) achieved for TEM-1 and PSE-4 could not be attained for the chimera cTEM-17m, which yielded 91% completeness for the ^1^H_N_ and ^15^N resonances [26]. Along with the missing resonances for Ser70 and Ala237, as in both TEM-1 and PSE-4, unassigned regions of cTEM-17m included numerous active-site residues: the Asp131-Asn132 portion of the SDN loop, residues Asn214, Val216 through Leu221, and residues Lys234 through Arg244. Previous spin relaxation studies for TEM-1 and PSE-4 have proposed that conformational exchange (*i.e.* motions on the µs-ms timescale) may occur in and about these active-site regions [23]–[25], suggesting that the additional missing resonances in cTEM-17m might result from a conformational exchange process (µs-ms timescale) focused around the active site, which broadens resonances. Notably, unassigned residues 214, 217 and 234 are included among the 11 aforementioned residues that are invariable in TEM-1 yet differ between TEM-1 and PSE-4 [52]. They are within the active-site area of cTEM-17m where regions originating from TEM-1 and PSE-4 meet and structural impact of those substitutions would be the greatest. This suggests that substitution of residues 214, 217 and 234 contributes to the altered local structure and/or slow time-scale dynamics.

To investigate the putative increase in active-site dynamics, longitudinal (R_1_) and transverse (R_2_) ^15^N spin relaxation rates as well as {^1^H}-^15^N NOE values were measured, providing insight into the dynamic character of cTEM-17m for the ps-ns and µs-ms timescales. From the high quality ^1^H-^15^N HSQC spectrum (Figure S4), it was possible to characterize amplitudes for 166 resolved peaks at 500 MHz and 600 MHz representing 73% of the 228 potentially observable amides. To ensure that multiple field relaxation data shares a high degree of consistency, data set consistency was tested through the use of the field-independent spectral density J(0) values [43], [55]. This consistency test determined that the spin-relaxation data was of good overall quality (Figure S6) and could be combined for model-free analysis.

Average R_1_, R_2_, and {^1^H}-^15^N NOE for cTEM-17m are shown in Table 4 along with previously reported values for the TEM-1 and PSE-4 parental proteins [23], [24]. The average backbone relaxation parameters for cTEM-17m were comparable to TEM-1 and PSE-4 at both fields studied. Residue-specific R_1_, R_2_, and {^1^H}-^15^N NOE relaxation data was plotted for cTEM-17m (Figure S5). As expected, the R_1_ data displayed a similar pattern at both fields (with R_1_^500^> R_1_^600^). Examination of the R_2_ data revealed regions possessing elevated R_2_ values, including residues in the 120–140 region, the recombined region (residues 150–190), and residues within the *C*-terminal portion of cTEM-17m. Elevated R_2_ values are indicative of residues that may be undergoing conformational exchange on the µs-ms timescale (further discussed below).

**Table 4:** Average backbone ^15^N spin relaxation parameters for TEM-1, cTEM-17m and PSE-4.

Variant	R_1_ (s^−1^)		R_2_ (s^−1^)		NOE	
	500 MHz	600 MHz	500 MHz	600 MHz	500 MHz	600 MHz
TEM-1^a^	1.33±0.07	1.04±0.03	16.0±0.8	17.1±0.6	0.75±0.04	0.80±0.04
cTEM-17m^b^	1.38±0.07	1.07±0.06	15.8±1.6	17.3±1.9	0.79±0.06	0.82±0.06
PSE-4^c^	1.34±0.08	0.99±0.07	15.6±1.4	16.5±1.6	0.78±0.05	0.80±0.06

a Values taken from [23].

b Spin relaxation statistics for residues with data at two magnetic fields (N = 167).

c Values taken from [24].

**Figure 3:**
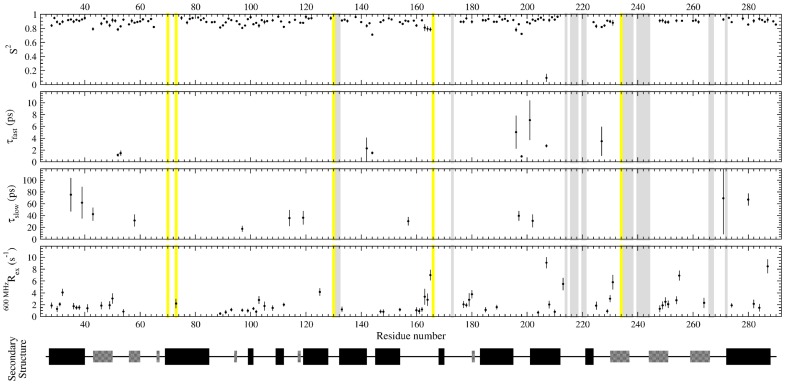
Model-free analysis for chimera cTEM-17m. Highlighted in yellow are active site residues (Ser70, Lys73, Ser130, Glue166 and Lys234). Unassigned residues are highlighted in grey. PSE-4 secondary structures are shown with helices as wide black boxes, and β-sheets as narrow grey boxes.

**Figure 4:**
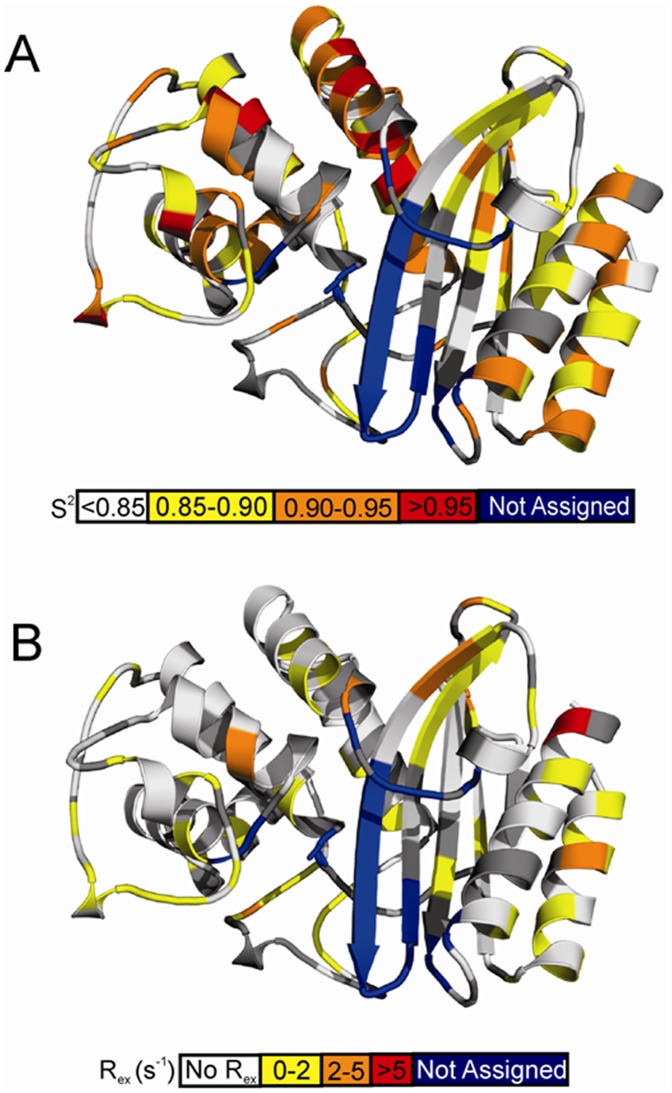
Motions extracted from the model-free analysis mapped on the homology model of cTEM-17m. A) Backbone amide ps-ns timescale generalized order parameter (S^2^). B) Conformational exchange term (R_ex_, model-free models *m3*, *m4*, *m7*, *m8*, and *m9*). Dark grey regions of cTEM-17m indicate residues without relaxation data due to either the presence of a proline residue or peak overlap. Blue regions indicate residues for which no backbone resonance could be assigned. The location of active-site Ser70 is circled.

To further correlate the relaxation data with the dynamics of cTEM-17m, analysis of relaxation data using the model-free formalism as implemented in the open source program “relax” was performed [41], [42], [56]–[58]. Model-free analysis provides information about the local and global dynamic character of a protein through the extraction of the order parameter (S^2^), describing the amplitude of local ps-ns timescale motions, conformational exchange (R_ex_) which accounts for motions on the µs-ms timescale, the local correlation time (τ_e_), and the global correlation time (τ_m_) of the protein [59] (Figure 3). cTEM-17m was best described by an ellipsoid diffusion tensor with D_||_/D⊥ and τ_m_ values similar to those of both TEM-1 and PSE-4 (Table 5 and Table S1). Following diffusion tensor optimization and model selection, the distribution of residues fitted to the various models (*m*) was as follows: *m0* (10), *m1* (78), *m2* (11), *m3* (50), *m4* (1), *m5* (7), *m6* (0), *m7* (1), *m8* (0), *m9* (8) for the 166 N-H vectors of cTEM-17m analyzed. Fitting for cTEM-17m was distributed more strongly in the complex models (≥ *m3*) compared to TEM-1 (*m0* (2), *m1* (68), *m2* (66), *m3* (12), *m4* (4), *m5* (21), *m6* (0), *m7* (2), *m8* (0), *m9* (2) for the 177 residues analyzed) and PSE-4 (*m0* (1), *m1* (129), *m2* (46), *m3* (28), *m4* (3), *m5* (19), *m6* (3), *m7* (0), *m8* (0), *m9* (1) for the 230 residues analyzed). This is most pronounced for model 3: TEM-1 (*m3* = 12 residues), PSE-4 (*m3* = 28) and cTEM-17 (*m3* = 50), indicating a greater distribution of the R_ex_ factor across cTEM-17m. Values for the model-free parameters obtained for cTEM-17m are represented in Figure 4 and are given in Table 5 along with the corresponding values for the parental TEM-1 and PSE-4.

**Table 5:** Model-free parameters for TEM-1, cTEM-17m and PSE-4.

Variant	<S^2^> d	D_||_/D_⊥_	τ_m_ (ns)
TEM-1^a^	0.89±0.05	1.23	12.4
cTEM-17m^b^	0.89±0.05	1.29	12.2
PSE-4^c^	0.87±0.05	1.32	12.7

a Values taken from [23].

b Minimized using an ellipsoid diffusion tensor.

c Values taken from [24].

d Values are given as the average +/− standard deviation from the mean.

The mean order parameter determined for cTEM-17m (<S^2^> = 0.89±0.05) was also found to be in excellent agreement with the published values for TEM-1 and PSE-4 (Table 5) indicating that the laboratory-evolved chimera cTEM-17m has retained a highly ordered structure, a hallmark of class A β-lactamases. In addition, the active site of cTEM-17m is also well ordered on the ps-ns timescale, as for TEM-1 and PSE-4 active sites, with many residues possessing S^2^ values ≥ 0.9 [23]–[25]. Overall, no region underwent large alterations in the mean order parameters for cTEM-17m relative to TEM-1 or PSE-4 (Figure 3). Interestingly, residue Glu64 located within the inter-domain linker region that connects the α- and α/β-domain of cTEM-17m, was found to be significantly more rigid (<S^2^> = 0.93±0.02) than the corresponding residue region in TEM-1 (<S^2^> = 0.87±0.01) and PSE-4 (<S^2^> = 0.89±0.01). Inspection of the homology model of cTEM-17m shows that Glu64 is predicted to lie in close proximity (within 5.6 Å) to the M182T mutation introduced as a result of recombination. The homology model predicts that the side chain of M182T is buried. This introduces the potential for new hydrogen bonding between the Thr182 hydroxyl and the backbone carbonyl oxygen (HOη_182_−O_66_) and nitrogen (HN_66_−O_182_) of Phe66, such that the M182T replacement may play a role in stabilizing the inter-domain linker region of cTEM-17m.

Previous NMR dynamics studies have found Tyr105 in both TEM-1 and PSE-4 to be one of the most flexible residues on a fast timescale, with S^2^ = 0.80±0.02 for both enzymes [23], [24]. This residue plays a role in substrate recognition in class A β-lactamases [60]. Previous structural and molecular dynamics simulated annealing studies have shown that the TEM-1 Tyr105 side-chain undergoes dynamic exchange between a subset of favoured rotamers [61], which may contribute to active-site stabilization in conjunction with the ‘SDN’ loop region and Val216, and may help orient the substrate within the active site [29]. Here, model-free analysis found Tyr105 of cTEM-17m to be significantly more rigid on the fast timescale than for its parents, with S^2^ = 0.89±0.02. The observed increase in fast timescale S^2^, along with the inclusion of R_ex_ in the model-free fitting for cTEM-17m Tyr105 (Table 6), suggests that the dynamic motion of this residue has shifted to a slower timescale. Tyr105 is located within a surface loop; examination of S^2^ for the flanking residues Glu104 and Ser106 shows that they are also more rigid, with S^2^ = 0.92±0.01 and 0.90±0.01, respectively (TEM-1: Glu104 S^2^ = 0.86±0.02 and Ser106 S^2^ = 0.81±0.02; PSE-4: Ser106 S^2^ = 0.83±0.01). This perturbation of the side-chain motions of Tyr105 may be linked to the slower timescale motions of the Asp131-Asn132 portion of the SDN loop and Val216 (as evidenced through exchange broadening), and may involve water molecules [29]. Nonetheless, this change is clearly tolerated both from a structural (NMR and CD) and functional (kinetics) standpoint.

**Table 6:** Residues in TEM-1, cTEM-17m and PSE-4 β-lactamases requiring the R_ex_ term in the model-free analysis.

Variant	Residues
TEM-1	Glu^28^, Leu^57^, Leu^76^, Ile^127^, Asp^131^, Leu^169^, Asn^170^, Glu^212^, Asp^214^, Ala^217^, Gly^218^, Leu^220^, Leu^221^, Ala^232^, Lys^234^, Gly^236^, Gly^245^, Gly^251^, Asp^254^, Gly^255^
PSE-4	Ala^35^, Trp^57^, Ala^76^, Ala^78^, Ser^93^, Glu^110^, Leu^119^, Asp^120^, Cys^123^, Met^127^, Thr^128^, Thr^133^, Asn^136^, Ile^138^, Val^148^, Leu^152^, Arg^153^, Arg^161^, Arg^178^, Leu^207^, Leu^220^, Leu^221^, Asn^230^, Asp^233^, Arg^234^, Ser^235^, Gly^236^, Arg^244^, Leu^265^, Asn^276^, Lys^281^, Ile^286^
cTEM-17m	Glu^28^, Leu^30^, Val^31^, Lys^32^, Ala^36^, Glu^37^, Asp^38^, Gly^41^, Tyr^46^, Leu^49^, Asp^50^, Gly^54^, Lys^73^, Gly^89^, Leu^91^, Arg^93^, Tyr^97^, Glu^99^, Asp^101^, Leu^102^, Val^103^, Tyr^105^, Val^108^, His^112^, Ala^125^, Thr^133^, Gly^147^, Leu^148^, Glu^154^, Thr^160^, Arg^161^, Leu^162^, Asp^163^, Arg^164^, Ile^165^, Leu^177^, Arg^178^, Asp^179^, Thr^180^, Ala^185^, Thr^189^, Arg^204^, Ile^208^, Trp^210^, Ala^213^, Leu^225^, Trp^229^, Phe^230^, Ile^231^, Ala^248^, Ala^249^, Leu^250^, Gly^251^, Asp^254^, Gly^255^, Tyr^264^, Gly^274^, Ile^282^, Ala^284^, Ile^287^

The recombined region in cTEM-17m (residues 150–190) has a mean <S^2^> = 0.89±0.03, closely matched to the values for the corresponding region of TEM-1 (<S^2^> = 0.90±0.03) and PSE-4 (<S^2^> = 0.88±0.04). While the segments comprising residues 150–161 and 177–190 were well ordered, with mean order parameters in very good agreement with the corresponding regions of TEM-1 and PSE-4, residues 162–176 have an <S^2^> = 0.84±0.05, which is significantly lower than the same region in TEM-1 (<S^2^> = 0.89±0.05) or PSE-4 (<S^2^> = 0.88±0.04). This region includes most of the Ω-loop (residues 161 through 179). Most significant is the reduced <S^2^> = 0.80 for residues 163–165, at the *N*-terminus of the Ω-loop. (<S^2^> in PSE-4 = 0.84 and in TEM-1 = 0.86); the tip of the Ω-loop (residues 166–175) could not be assigned in cTEM-17m due to peak overlap or broadening. This suggests that the catalytically relevant Ω-loop of the chimera has an altered dynamic character, with greater flexibility than the parental TEM-1 and PSE-4 on the ps-ns timescale. We note that the Ω-loop is spatially adjacent to the series of unassigned residues 234–244 in cTEM-17m, suggesting that the new dynamics inferred for region 234–244 may be linked to those of the Ω-loop. Recent molecular dynamics simulations of TEM-1 proposed that the tip of the Ω-loop (residues 173–177) is flexible on the 50 ns time-scale [25], [62]. While this was not experimentally observed upon NMR investigation of TEM-1 [23], the greater flexibility on the fast timescale observed for some residues of the cTEM-17m Ω-loop suggests that structure-based recombination has altered the fast timescale motions of the remainder of the loop.

**Figure 5:**
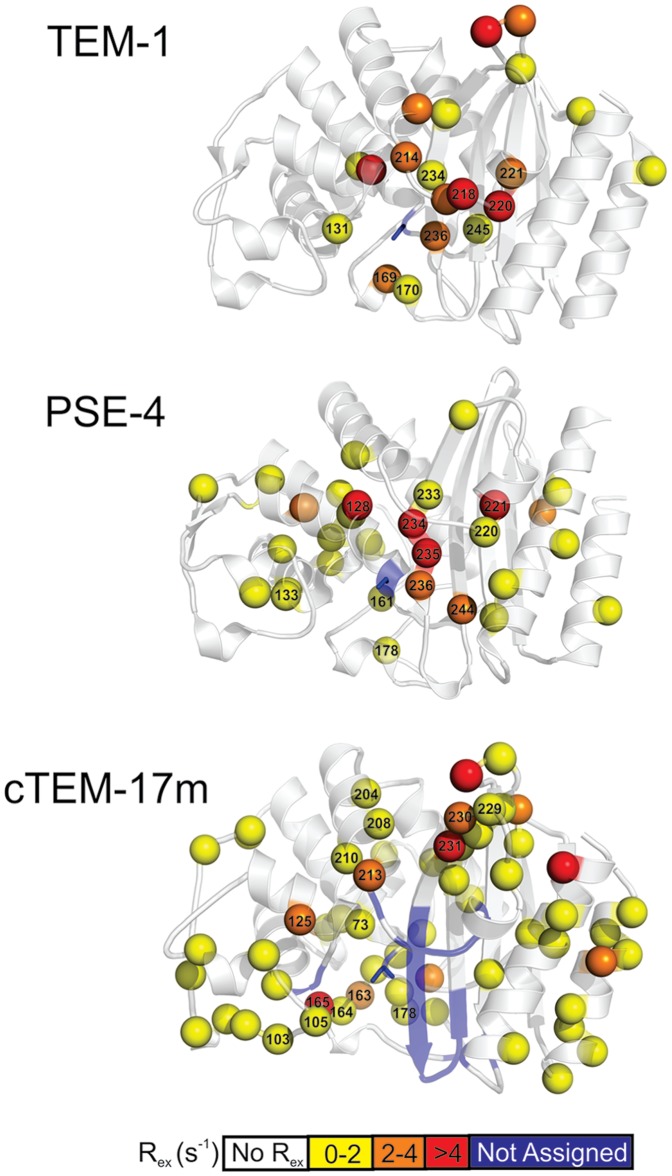
Comparison of slow motions for the parental and chimeric enzymes. Residues for which the conformational exchange term R_ex_ was extracted from model-free models *m3*, *m4*, *m7*, *m8*, or *m9*, indicative of dynamics on the µs-ms timescale, were scaled to the same field (600 MHz) and mapped as spheres for TEM-1 [23], PSE-4 [24] and cTEM-17m. The spheres are colored according to the magnitude of R_ex,_ as defined in the scale below. Residues for which backbone NMR assignments are missing, potentially indicating the presence of µs-ms motions, are in blue. The active-site serine is in sticks representation.

A greater number of residues in cTEM-17m required model-free fitting with an R_ex_ term (*i.e.* more residues fitted with models 3, 4, 7 and 9) than for TEM-1 and PSE-4 (Table 6). R_ex_ can be related to local conformational exchange, and thus to dynamics on the slower, µs-ms time-scale. Twenty and 32 residues required fitting to a model with a R_ex_ term for the parental TEM-1 and PSE-4, respectively, *versus* 61 residues for the cTEM-17m chimera (∼37% of residues probed using this approach, Figure 5). This indicates that significantly more residues within cTEM-17m undergo slow µs-ms timescale motions relative to either parental protein. Those residues are dispersed throughout the structure. The turn connecting β-strands 4 and 5 displayed a slight extension in the number of residues requiring fitting with the R_ex_ term. Specifically, TEM-1 residues 251, 254 and 255 required fitting with an R_ex_ term, suggesting some dynamic potential to the region, though no R_ex_ term was required to fit the same region in PSE-4; in chimera cTEM-17m, the same 3 residues (251, 254, 255) as well as β-strand residues 248–250 all required fitting with an R_ex_ term. We note that the region encompassing residues 234–244 in cTEM-17m is unassigned, suggesting increased dynamics on the slow timescale; several residues required an R_ex_ term in that region for both TEM-1 and PSE-4 (Figure 5), consistent with some dynamic behaviour in that area of the parental enzymes. Interestingly, an important number of residues located within the 150–190 segment of cTEM-17m required fitting with the R_ex_ term (Figures 3, 5 and Table S2), suggesting that the PSE-4-derived region of cTEM-17m may undergo increased µs-ms motions. It has been speculated that the Ω-loop region (residues 161 to 179) of class A β-lactamases undergo slow motions based on crystal structures and molecular modeling [21], [25], but this remains controversial. Previous model-free analysis of TEM-1 and PSE-4 predicted that Ω-loop residues in TEM-1 (Arg164, Leu166 and Leu169) and PSE-4 (Arg161 and Arg178) exhibit slow µs-ms conformational exchange and potentially high-amplitude motions with hinge points that allow for movement of the Ω-loop in PSE-4 [23]–[25]. The potential for a slow, flap-like motion of the Ω-loop region has clear implications in terms of catalysis since this motion would re-position Glu166 proximal to the catalytic Ser70, thus allowing Glu166 to participate in both the acylation and deacylation steps of the catalytic mechanism [24], [63]. Here, among other motions observed, conformational exchange was predicted for residues in the 160–162 and 177–179 “hinge” regions of cTEM-17m, suggesting that the cTEM-17m Ω-loop may conserve a PSE-4-like flexible nature on the µs-ms timescale (**Table S2**). Experiments are ongoing to further probe this effect.

### Conclusion

We have analyzed the activity and stability of three laboratory-generated chimeric β-lactamases derived from homologous class A β-lactamases: TEM-1 and PSE-4. Despite harbouring highly blended active sites, the chimeras display the broad substrate recognition profiles and catalytic efficiencies that are generally comparable to that of the parental enzymes. The function of each chimeric enzyme was most similar to the parental enzyme to which it is most closely related. Nonetheless, significant increases and decreases in catalytic efficiency were observed, such that the more highly substituted chimeras display a substrate spectrum distinct from the parental enzymes. Remarkably, the thermal stability of two out of the three chimeras was native-like, despite the high number of substitutions they include. This is doubtless a reflection of the non-random nature of those substitutions, which are the result of structure-based recombination between homologues. Moreover, the high ps-ns timescale order that characterizes TEM-1 and PSE-4 β-lactamases was conserved in chimera cTEM-17m. However, a greater number of µs-ms motions were predicted for cTEM-17m by model-free analyses of NMR resonances, in agreement with the greater number of residues in the vicinity of the active site for which resonances cannot be observed. Our results lend further support to the use of structure-guided protein recombination by SCHEMA to obtain well-folded and active chimeras that include high sequence diversity, and thus allow for exploration of functional modulation.

## Supporting Information

Figure S1 **β-Lactam substrates used in this work**
(DOC)

Figure S2 **Michaelis-Menten analysis of carbenicillin hydrolysis, monitored in 10 cm path-length cells.** Representative Michaelis-Menten analysis of carbenicillin (CB) hydrolysis is shown for each of the β-lactamases for which this has not previously been reported: the parental PSE-4 (red) and the chimeras cTEM-17m (gold), cTEM-67m (green) and cTEM-92m (black). As a result of its low molar absorption coefficient, hydrolysis of carbenicillin was monitored in 10 cm path-length cells.
(DOC)

Figure S3 **Specific activity of the chimera cTEM-17m at 0.8 mM monitored over a one-week period at 31.5 °C under the NMR sample conditions.**
(DOC)

Figure S4 **2D ^1^H-^15^N-HSQC of [^15^N/^13^C]-labeled cTEM-17m at a protein concentration of 0.4 mM (recorded at 11.7 T, pH = 6.8, 31.5°C) (Morin, Clouthier et al. 2010).**
(DOC)

Figure S5 **cTEM-17m ^15^N spin relaxation parameters (R_1_, R_2_, R2/R1, and {^1^H}-^15^N-NOE) obtained at 500 (red) and 600 MHz (blue).** Relaxation data located within the 150 to 190 chimeral sequence exchange region of cTEM-17m is shown (black square). Grey shaded areas represent regions of cTEM-17m where complete backbone assignments could not be obtained, while yellow shaded areas represent the locations of the catalytically relevant residues Ser70, Lys73, Ser130, Glu166, and Arg244. PSE-4 secondary structures (highly similar to those for TEM-1) are shown with helices as wide black boxes, and β sheets as narrow grey boxes.
(DOC)

Figure S6 **Consistency test results as based on the method of Morin and Gagné (Morin, S. and Gagné S.M., J Biomol NMR, 2009. 45(4): 361-72).** J(0) values are compared for datasets acquired at 500 and 600 MHz (top: correlation plot; bottom: distribution plot of the ratios, with the mean values and standard deviations indicated). Data are shown for all residues (in black), for residues not fitted with a R_ex_ term during model-free analysis (in blue), and for residues with a R_ex_ term (*i.e.* affected by µs-ms motions, in red). Most outliers are residues fitted with a R_ex_ parameter. When excluding these residues (since R_ex_ is quadratically dependent on the magnetic field, and the J(0) test is not valid for such residues), the magnetic field independent function J(0) yields very similar values for both data recorded at 500 and 600 MHz. This indicates high consistency of both sets of data.
(DOC)

Figure S7 **cTEM-17m diffusion tensor and distribution of N-H vectors used to characterize it.** Represented are views of the ellipsoid diffusion tensor along its three principal axes. N-H vectors orientations are shown as surface on the tip of artificial vectors of length 20 Å placed at the centre of mass of the protein. These vectors are duplicated in the opposite direction because of symmetry of the ellipsoidal diffusion tensor.
(DOC)

Table S1 **cTEM-17m ^15^N spin relaxation data.**
(PDF)

Table S2 **cTEM-17m model-free parameters.**
(PDF)

Supporting Information S1 (DOC)
