# Supplementary material for: Chimeric β-Lactamases: Global Conservation of Parental Function and Fast Time-Scale Dynamics with Increased Slow Motions
Source: PLoS One. 2012 Dec 21;7(12):e52283. doi: 10.1371/journal.pone.0052283 (PMC3528772; doi:10.1371/journal.pone.0052283)
Supplement: Figure S2 — Michaelis-Menten analysis of carbenicillin hydrolysis, monitored in 10 cm path-length cells. Representative Michaelis-Menten analysis of carbenicillin (CB) hydrolysis is shown for each of the β-lactamases for which this has not previously been reported: the parental PSE-4 (red) and the chimeras cTEM-17m (gold), cTEM-67m (green) and cTEM-92m (black). As a result of its low molar absorption coefficient, hydrolysis of carbenicillin was monitored in 10 cm path-length cells. (DOC) [file pone.0052283.s002.doc]

F**igure S2:**
