# Supplementary figures and images for: Chimeric β-Lactamases: Global Conservation of Parental Function and Fast Time-Scale Dynamics with Increased Slow Motions
Source: PLoS One. 2012 Dec 21;7(12):e52283. doi: 10.1371/journal.pone.0052283 (PMC3528772; doi:10.1371/journal.pone.0052283)

**Figure S3**:


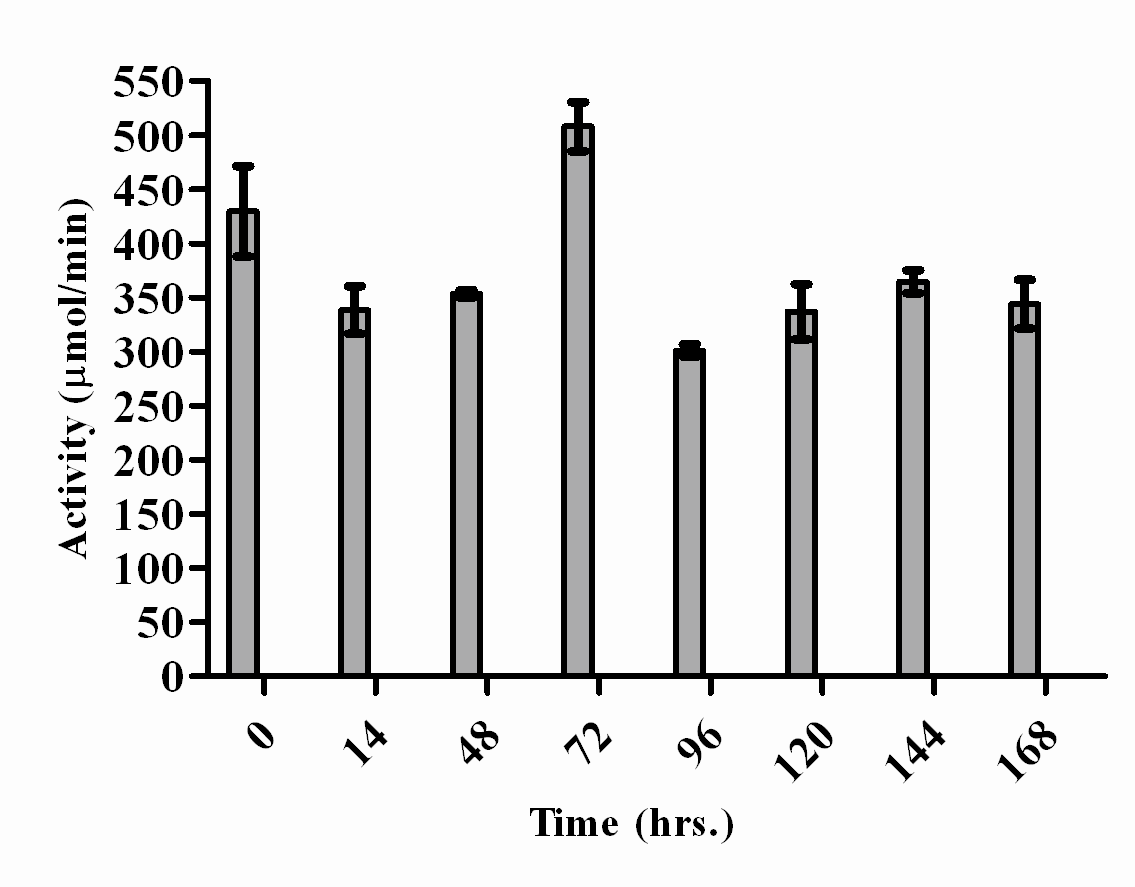

Supplement: Figure S3 — Specific activity of the chimera cTEM-17m at 0.8 mM monitored over a one-week period at 31.5 °C under the NMR sample conditions. (DOC) [file pone.0052283.s003.doc]

**Figure S4:**


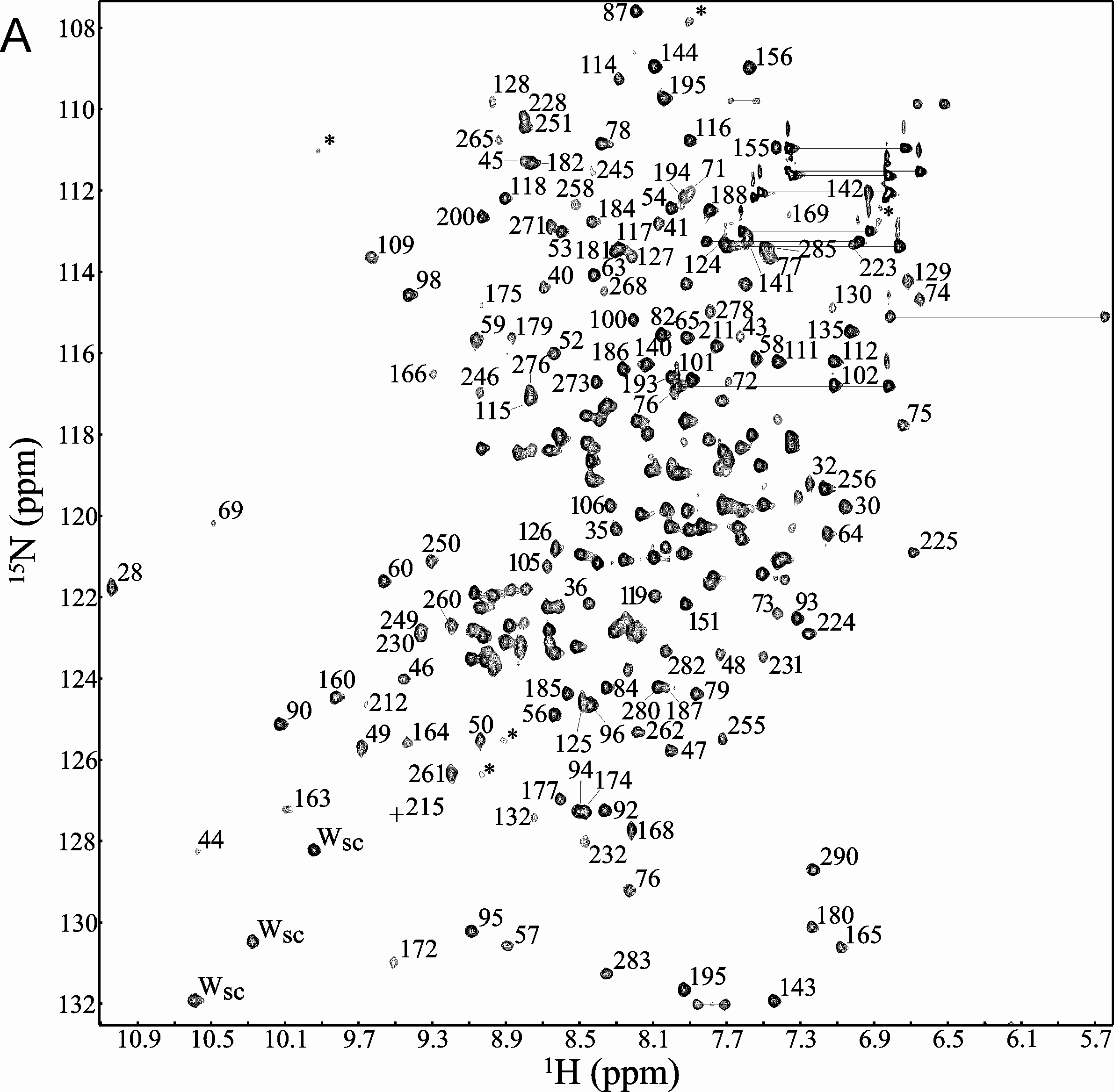

Supplement: Figure S4 — 2D 1H-15N-HSQC of [15N/13C]-labeled cTEM-17m at a protein concentration of 0.4 mM (recorded at 11.7 T, pH = 6.8, 31.5°C) (Morin, Clouthier et al. 2010). (DOC) [file pone.0052283.s004.doc]

*
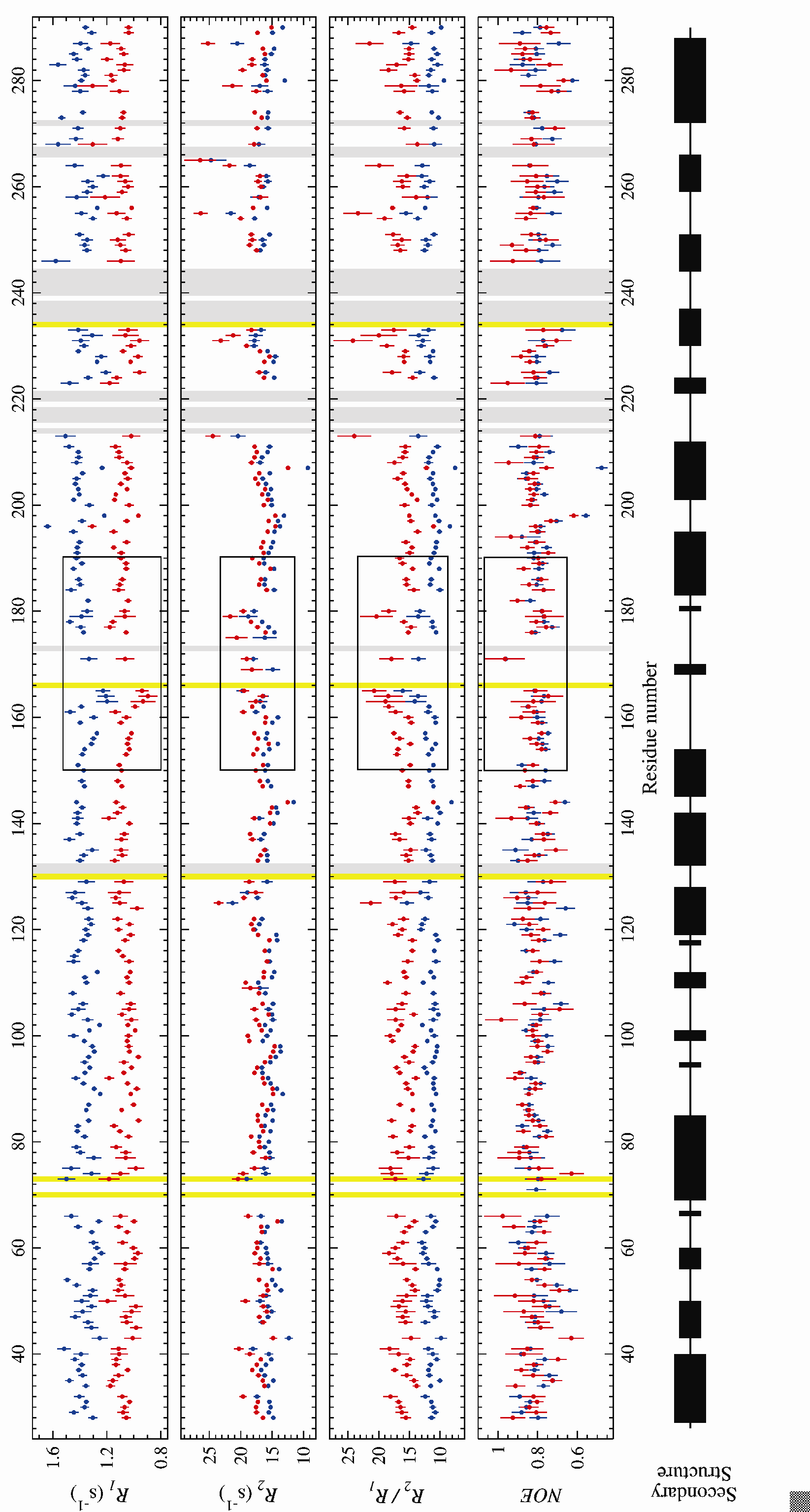
*

Supplement: Figure S5 — cTEM-17m 15N spin relaxation parameters (R1, R2, R2/R1, and {1H}-15N-NOE) obtained at 500 (red) and 600 MHz (blue). Relaxation data located within the 150 to 190 chimeral sequence exchange region of cTEM-17m is shown (black square). Grey shaded areas represent regions of cTEM-17m where complete backbone assignments could not be obtained, while yellow shaded areas represent the locations of the catalytically relevant residues Ser70, Lys73, Ser130, Glu166, and Arg244. PSE-4 secondary structures (highly similar to those for TEM-1) are shown with helices as wide black boxes, and β sheets as narrow grey boxes. (DOC) [file pone.0052283.s005.doc]
