# Supplementary material for: Chimeric β-Lactamases: Global Conservation of Parental Function and Fast Time-Scale Dynamics with Increased Slow Motions
Source: PLoS One. 2012 Dec 21;7(12):e52283. doi: 10.1371/journal.pone.0052283 (PMC3528772; doi:10.1371/journal.pone.0052283)
Supplement: Figure S6 — Consistency test results as based on the method of Morin and Gagné (Morin, S. and Gagné S.M., J Biomol NMR, 2009. 45(4): 361-72). J(0) values are compared for datasets acquired at 500 and 600 MHz (top: correlation plot; bottom: distribution plot of the ratios, with the mean values and standard deviations indicated). Data are shown for all residues (in black), for residues not fitted with a Rex term during model-free analysis (in blue), and for residues with a Rex term (i.e. affected by µs-ms motions, in red). Most outliers are residues fitted with a Rex parameter. When excluding these residues (since Rex is quadratically dependent on the magnetic field, and the J(0) test is not valid for such residues), the magnetic field independent function J(0) yields very similar values for both data recorded at 500 and 600 MHz. This indicates high consistency of both sets of data. (DOC) [file pone.0052283.s006.doc]

**Figure S6**:

*
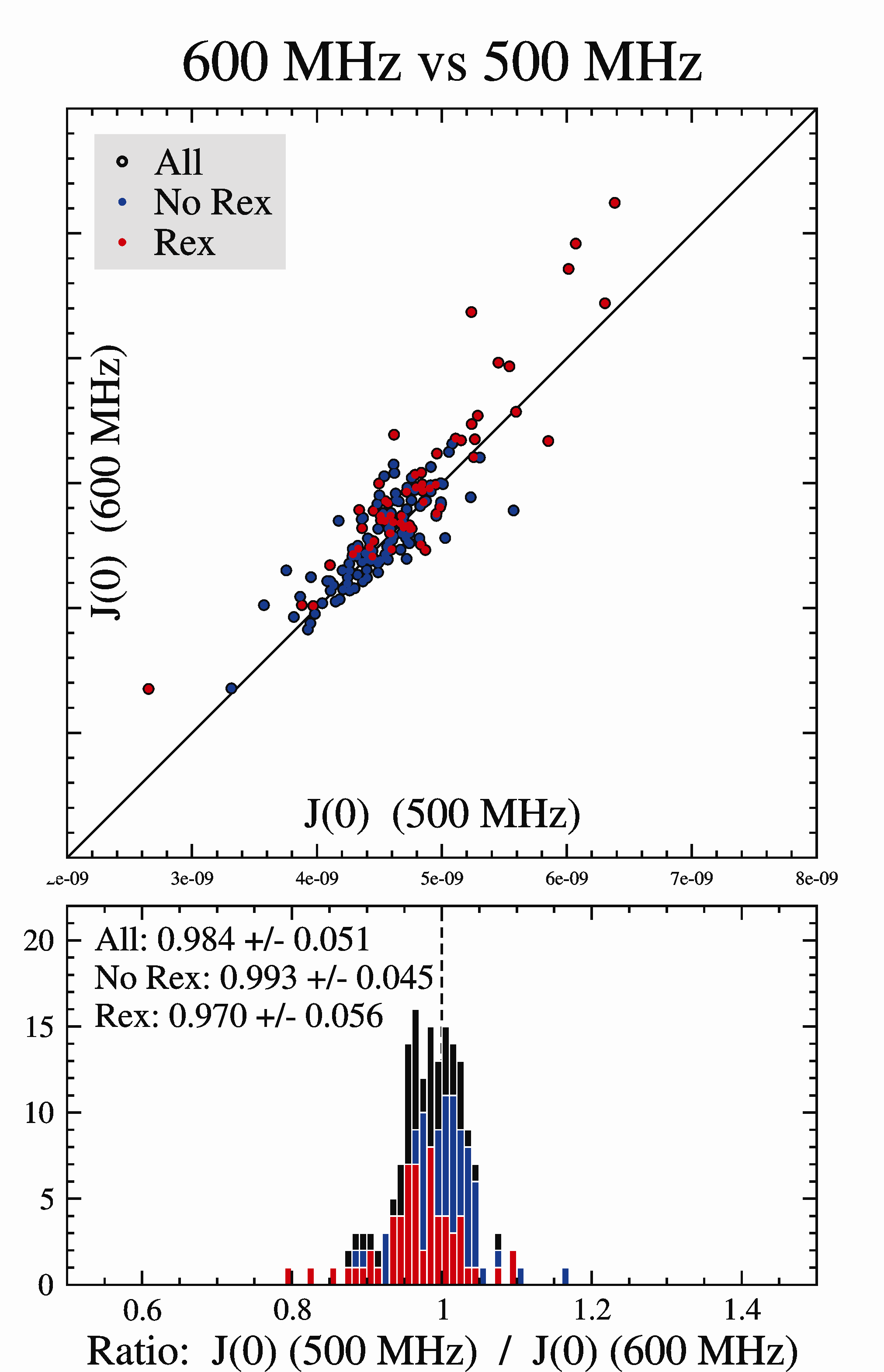
*

**Figure S7:** cTEM-17m diffusion tensor and distribution of N-H vectors used to characterise it. Represented are views of the ellipsoid diffusion tensor along its three principal axes. N-H vectors orientations are shown as surface on the tip of artificial vectors of length 20 Å placed at the centre of mass of the protein. These vectors are duplicated in the opposite direction because of symmetry of the ellipsoidal diffusion tensor.
