# Supplementary material for: Chimeric β-Lactamases: Global Conservation of Parental Function and Fast Time-Scale Dynamics with Increased Slow Motions
Source: PLoS One. 2012 Dec 21;7(12):e52283. doi: 10.1371/journal.pone.0052283 (PMC3528772; doi:10.1371/journal.pone.0052283)
Supplement: Figure S7 — cTEM-17m diffusion tensor and distribution of N-H vectors used to characterize it. Represented are views of the ellipsoid diffusion tensor along its three principal axes. N-H vectors orientations are shown as surface on the tip of artificial vectors of length 20 Å placed at the centre of mass of the protein. These vectors are duplicated in the opposite direction because of symmetry of the ellipsoidal diffusion tensor. (DOC) [file pone.0052283.s007.doc]

**Figure S7:**
