# Supplementary material for: Chimeric β-Lactamases: Global Conservation of Parental Function and Fast Time-Scale Dynamics with Increased Slow Motions
Source: PLoS One. 2012 Dec 21;7(12):e52283. doi: 10.1371/journal.pone.0052283 (PMC3528772; doi:10.1371/journal.pone.0052283)
Supplement: Table S1 — cTEM-17m 15N spin relaxation data. (PDF) [file pone.0052283.s008.pdf]

**Table S1:** cTEM-17m $^{15}$N spin relaxation data

| Residue | | 500 MHz | | | | | | 600 MHz | | | | | |
|---|---|---|---|---|---|---|---|---|---|---|---|---|---|
| # | aa | $R_1$ ($s^{-1}$) | $\Delta R_1$ ($s^{-1}$) | $R_2$ ($s^{-1}$) | $\Delta R_2$ ($s^{-1}$) | $NOE$ | $\Delta NOE$ | $R_1$ ($s^{-1}$) | $\Delta R_1$ ($s^{-1}$) | $R_2$ ($s^{-1}$) | $\Delta R_2$ ($s^{-1}$) | $NOE$ | $\Delta NOE$ |
| 26 | His | n-ter | n-ter | n-ter | n-ter | n-ter | n-ter | n-ter | n-ter | n-ter | n-ter | n-ter | n-ter |
| 27 | Pro | - | - | - | - | - | - | - | - | - | - | - | - |
| 28 | Glu | 1.303 | 0.034 | 14.814 | 0.395 | 0.798 | 0.044 | 1.057 | 0.034 | 16.460 | 0.392 | 0.925 | 0.062 |
| 29 | Thr | 1.444 | 0.037 | 15.532 | 0.405 | 0.881 | 0.049 | 1.079 | 0.042 | 17.495 | 0.439 | 0.807 | 0.053 |
| 30 | Leu | 1.365 | 0.032 | 15.241 | 0.372 | 0.857 | 0.040 | 1.069 | 0.032 | 17.556 | 0.382 | 0.841 | 0.046 |
| 31 | Val | 1.351 | 0.020 | 15.428 | 0.257 | 0.838 | 0.029 | 1.031 | 0.022 | 17.268 | 0.256 | 0.802 | 0.032 |
| 32 | Lys | 1.403 | 0.040 | 17.406 | 0.561 | 0.891 | 0.052 | 1.086 | 0.039 | 19.627 | 0.554 | 0.820 | 0.059 |
| 33 | Val | o.l. | o.l. | o.l. | o.l. | o.l. | o.l. | o.l. | o.l. | o.l. | o.l. | o.l. | o.l. |
| 34 | Lys | 1.355 | 0.026 | 15.618 | 0.311 | 0.771 | 0.033 | 1.174 | 0.035 | 16.214 | 0.281 | 0.912 | 0.049 |
| 35 | Asp | 1.478 | 0.032 | 14.800 | 0.376 | 0.723 | 0.040 | 1.155 | 0.040 | 16.470 | 0.313 | 0.726 | 0.050 |
| 36 | Ala | 1.379 | 0.033 | 16.193 | 0.413 | 0.741 | 0.043 | 1.113 | 0.041 | 17.168 | 0.449 | 0.823 | 0.055 |
| 37 | Glu | 1.408 | 0.020 | 16.731 | 0.269 | 0.817 | 0.026 | 1.046 | 0.022 | 18.178 | 0.256 | 0.882 | 0.036 |
| 38 | Asp | 1.385 | 0.026 | 15.986 | 0.331 | 0.807 | 0.035 | 1.132 | 0.030 | 17.443 | 0.334 | 0.819 | 0.044 |
| 39 | Gln | 1.437 | 0.032 | 15.138 | 0.399 | 0.765 | 0.043 | 1.129 | 0.037 | 16.809 | 0.352 | 0.698 | 0.045 |
| 40 | Leu | 1.391 | 0.055 | 15.532 | 0.708 | 0.883 | 0.073 | 1.109 | 0.063 | 18.559 | 0.806 | 0.870 | 0.095 |
| 41 | Gly | 1.516 | 0.050 | 18.045 | 0.690 | 0.836 | 0.056 | 1.113 | 0.057 | 20.289 | 0.770 | 0.852 | 0.082 |
| 42 | Ala | o.l. | o.l. | o.l. | o.l. | o.l. | o.l. | o.l. | o.l. | o.l. | o.l. | o.l. | o.l. |
| 43 | Arg | 1.253 | 0.058 | 12.336 | 0.633 | 0.630 | 0.062 | 1.008 | 0.063 | 14.847 | 0.610 | 0.631 | 0.064 |
| 44 | Val | o.l. | o.l. | o.l. | o.l. | o.l. | o.l. | o.l. | o.l. | o.l. | o.l. | o.l. | o.l. |
| 45 | Gly | 1.314 | 0.051 | o.l. | o.l. | 0.786 | 0.065 | 0.982 | 0.046 | o.l. | o.l. | 0.785 | 0.061 |
| 46 | Tyr | 1.339 | 0.040 | 16.587 | 0.582 | 0.815 | 0.050 | 1.051 | 0.046 | 16.430 | 0.484 | 0.798 | 0.060 |
| 47 | Ile | 1.434 | 0.039 | 15.671 | 0.405 | 0.812 | 0.043 | 1.059 | 0.045 | 17.046 | 0.454 | 0.829 | 0.060 |
| 48 | Glu | 1.378 | 0.064 | 15.085 | 0.662 | 0.681 | 0.078 | 1.016 | 0.068 | 15.838 | 0.599 | 0.870 | 0.103 |
| 49 | Leu | 1.313 | 0.040 | 15.697 | 0.570 | 0.740 | 0.053 | 0.984 | 0.050 | 16.417 | 0.544 | 0.760 | 0.068 |
| 50 | Asp | 1.386 | 0.057 | 16.918 | 0.716 | 0.770 | 0.064 | 1.195 | 0.066 | 19.252 | 0.780 | 0.820 | 0.079 |
| 51 | Leu | 1.322 | 0.055 | 15.928 | 0.670 | 0.818 | 0.069 | 1.065 | 0.070 | 16.442 | 0.744 | 0.914 | 0.104 |
| 52 | Asn | 1.304 | 0.036 | 13.591 | 0.436 | 0.639 | 0.041 | 1.115 | 0.041 | 15.698 | 0.383 | 0.691 | 0.049 |
| 53 | Ser | 1.423 | 0.032 | 14.471 | 0.332 | 0.703 | 0.033 | 1.094 | 0.031 | 15.873 | 0.294 | 0.765 | 0.041 |
| 54 | Gly | 1.493 | 0.027 | 15.010 | 0.302 | 0.804 | 0.027 | 1.108 | 0.027 | 17.082 | 0.290 | 0.829 | 0.038 |
| 55 | Lys | o.l. | o.l. | o.l. | o.l. | o.l. | o.l. | o.l. | o.l. | o.l. | o.l. | o.l. | o.l. |
| 56 | Ile | 1.328 | 0.023 | 13.884 | 0.250 | 0.830 | 0.036 | 1.067 | 0.026 | 14.924 | 0.231 | 0.765 | 0.035 |
| 57 | Leu | 1.323 | 0.063 | 15.673 | 0.871 | 0.740 | 0.079 | 1.063 | 0.084 | 17.036 | 1.033 | 0.894 | 0.119 |
| 58 | Glu | 1.291 | 0.023 | 15.678 | 0.367 | 0.752 | 0.033 | 0.994 | 0.028 | 16.844 | 0.282 | 0.761 | 0.041 |
| 59 | Ser | 1.240 | 0.029 | 15.846 | 0.430 | 0.758 | 0.043 | 0.970 | 0.035 | 17.771 | 0.429 | 0.864 | 0.060 |
| 60 | Phe | 1.274 | 0.026 | 15.978 | 0.355 | 0.864 | 0.034 | 1.004 | 0.029 | 17.349 | 0.315 | 0.849 | 0.042 |
| 61 | Arg | 1.299 | 0.035 | 16.845 | 0.526 | 0.897 | 0.049 | 1.083 | 0.040 | 17.420 | 0.479 | 0.806 | 0.052 |
| 62 | Pro | - | - | - | - | - | - | - | - | - | - | - | - |
| 63 | Glu | 1.311 | 0.024 | 16.151 | 0.343 | 0.828 | 0.033 | 1.049 | 0.027 | 16.623 | 0.264 | 0.768 | 0.036 |
| 64 | Glu | 1.413 | 0.030 | 15.758 | 0.360 | 0.816 | 0.038 | 1.112 | 0.034 | 16.744 | 0.341 | 0.920 | 0.057 |
| 65 | Arg | 1.258 | 0.026 | 13.474 | 0.277 | 0.816 | 0.033 | 0.998 | 0.027 | 14.137 | 0.222 | 0.788 | 0.038 |
| 66 | Phe | 1.463 | 0.054 | 16.799 | 0.672 | 0.752 | 0.064 | 1.099 | 0.056 | 18.811 | 0.713 | 0.976 | 0.093 |
| 67 | Pro | - | - | - | - | - | - | - | - | - | - | - | - |
| 68 | Met | o.l. | o.l. | o.l. | o.l. | o.l. | o.l. | o.l. | o.l. | o.l. | o.l. | o.l. | o.l. |
| 69 | Met | o.l. | o.l. | o.l. | o.l. | o.l. | o.l. | o.l. | o.l. | o.l. | o.l. | o.l. | o.l. |
| **70** | **Ser** | **n.o.** | **n.o.** | **n.o.** | **n.o.** | **n.o.** | **n.o.** | **n.o.** | **n.o.** | **n.o.** | **n.o.** | **n.o.** | **n.o.** |
| 71 | Thr | o.l. | o.l. | o.l. | o.l. | 0.807 | 0.049 | o.l. | o.l. | o.l. | o.l. | o.l. | o.l. |
| 72 | Phe | o.l. | o.l. | o.l. | o.l. | o.l. | o.l. | o.l. | o.l. | o.l. | o.l. | o.l. | o.l. |
| **73** | **Lys** | **1.499** | **0.066** | **19.039** | **0.901** | **0.798** | **0.067** | **1.183** | **0.079** | **20.441** | **0.943** | **0.781** | **0.074** |
| 74 | Val | 1.313 | 0.065 | 16.016 | 0.778 | o.l. | o.l. | 1.099 | 0.066 | 19.606 | 0.853 | 0.629 | 0.061 |
| 75 | Leu | 1.465 | 0.067 | 16.283 | 0.792 | 0.841 | 0.074 | 0.985 | 0.062 | 17.814 | 0.772 | 0.794 | 0.073 |
| 76 | Leu | o.l. | o.l. | o.l. | o.l. | o.l. | o.l. | o.l. | o.l. | o.l. | o.l. | o.l. | o.l. |
| 77 | Cys | 1.297 | 0.056 | 15.323 | 0.713 | 0.835 | 0.071 | 1.060 | 0.075 | 16.047 | 0.862 | 0.893 | 0.111 |
| 78 | Gly | 1.396 | 0.034 | 15.414 | 0.446 | 0.841 | 0.046 | 1.059 | 0.041 | 17.989 | 0.481 | 0.892 | 0.060 |
| 79 | Ala | 1.426 | 0.036 | 16.207 | 0.465 | 0.870 | 0.048 | 1.130 | 0.043 | 16.944 | 0.416 | 0.856 | 0.063 |
| 80 | Val | o.l. | o.l. | 15.517 | 0.186 | o.l. | o.l. | o.l. | o.l. | 17.088 | 0.163 | o.l. | o.l. |
| 81 | Leu | 1.364 | 0.027 | 17.004 | 0.340 | 0.793 | 0.030 | 1.038 | 0.028 | 18.332 | 0.326 | 0.758 | 0.040 |
| 82 | Ser | 1.418 | 0.021 | 15.247 | 0.255 | 0.751 | 0.026 | 1.103 | 0.024 | 16.423 | 0.223 | 0.872 | 0.037 |
| 83 | Arg | 1.415 | 0.024 | 16.171 | 0.324 | 0.878 | 0.034 | 1.146 | 0.029 | 16.705 | 0.297 | 0.789 | 0.037 |
| 84 | Val | 1.334 | 0.022 | 14.920 | 0.290 | 0.796 | 0.032 | 0.964 | 0.025 | 17.259 | 0.291 | 0.825 | 0.039 |
| 85 | Asp | o.l. | o.l. | 16.056 | 0.193 | 0.816 | 0.021 | o.l. | o.l. | 17.301 | 0.183 | 0.844 | 0.025 |
| 86 | Ala | 1.352 | 0.016 | 14.822 | 0.181 | 0.851 | 0.023 | 1.090 | 0.019 | 15.740 | 0.165 | 0.844 | 0.028 |
| 87 | Gly | 1.334 | 0.018 | 15.143 | 0.217 | 0.844 | 0.024 | 1.002 | 0.021 | 16.567 | 0.203 | 0.878 | 0.031 |
| 88 | Gln | o.l. | o.l. | o.l. | o.l. | o.l. | o.l. | o.l. | o.l. | o.l. | o.l. | o.l. | o.l. |
| 89 | Glu | 1.249 | 0.012 | 13.310 | 0.165 | 0.845 | 0.021 | 1.022 | 0.014 | 14.841 | 0.139 | 0.844 | 0.022 |
| 90 | Gln | 1.294 | 0.021 | 14.215 | 0.285 | 0.841 | 0.030 | 0.978 | 0.025 | 14.921 | 0.229 | 0.812 | 0.037 |
| 91 | Leu | 1.373 | 0.022 | 15.221 | 0.257 | 0.784 | 0.027 | 1.047 | 0.022 | 16.241 | 0.243 | 0.810 | 0.031 |
| 92 | Gly | 1.430 | 0.031 | 15.653 | 0.362 | 0.833 | 0.032 | 1.183 | 0.035 | 16.471 | 0.314 | 0.914 | 0.045 |
| 93 | Arg | 1.362 | 0.019 | 16.546 | 0.242 | 0.883 | 0.027 | 1.074 | 0.020 | 17.804 | 0.228 | 0.892 | 0.031 |
| 94 | Arg | 1.327 | 0.020 | 16.649 | 0.461 | o.l. | o.l. | 1.017 | 0.022 | 17.373 | 0.237 | o.l. | o.l. |
| 95 | Ile | 1.364 | 0.030 | 15.275 | 0.366 | 0.798 | 0.035 | 1.073 | 0.037 | 16.151 | 0.390 | 0.814 | 0.048 |
| 96 | His | 1.334 | 0.021 | 14.432 | 0.240 | 0.801 | 0.029 | 0.966 | 0.022 | 15.260 | 0.203 | 0.835 | 0.034 |
| 97 | Tyr | 1.293 | 0.019 | 13.638 | 0.203 | 0.751 | 0.026 | 1.032 | 0.020 | 14.828 | 0.172 | 0.751 | 0.030 |
| 98 | Ser | 1.307 | 0.025 | 13.686 | 0.293 | 0.748 | 0.033 | 1.037 | 0.029 | 14.604 | 0.266 | 0.801 | 0.040 |

**Table S1:** cTEM-17m $^{15}$N spin relaxation data (continued)

| Residue | | 500 MHz | | | | | | 600 MHz | | | | | |
|---|---|---|---|---|---|---|---|---|---|---|---|---|---|
| # | aa | $R_1$ | $\Delta R_1$ | $R_2$ | $\Delta R_2$ | $NOE$ | $\Delta NOE$ | $R_1$ | $\Delta R_1$ | $R_2$ | $\Delta R_2$ | $NOE$ | $\Delta NOE$ |
| | | ($s^{-1}$) | ($s^{-1}$) | ($s^{-1}$) | ($s^{-1}$) | | | ($s^{-1}$) | ($s^{-1}$) | ($s^{-1}$) | ($s^{-1}$) | | |
| 99 | Gln | 1.367 | 0.019 | 16.491 | 0.263 | 0.813 | 0.024 | 1.049 | 0.018 | 18.631 | 0.231 | 0.798 | 0.026 |
| 100 | Asn | 1.446 | 0.039 | 15.817 | 0.451 | 0.755 | 0.033 | 1.039 | 0.029 | 18.861 | 0.374 | 0.822 | 0.040 |
| 101 | Asp | 1.329 | 0.016 | 15.223 | 0.183 | 0.859 | 0.023 | 0.990 | 0.016 | 16.648 | 0.159 | 0.825 | 0.027 |
| 102 | Leu | 1.253 | 0.017 | 16.141 | 0.274 | 0.822 | 0.027 | 1.044 | 0.020 | 17.024 | 0.198 | 0.807 | 0.030 |
| 103 | Val | 1.341 | 0.043 | 14.857 | 0.578 | 0.787 | 0.055 | 1.018 | 0.051 | 17.546 | 0.540 | 0.983 | 0.083 |
| 104 | Glu | 1.460 | 0.031 | 15.017 | 0.317 | 0.784 | 0.042 | 1.088 | 0.033 | 15.555 | 0.291 | 0.787 | 0.044 |
| **105** | **Tyr** | **1.411** | **0.054** | **15.581** | **0.620** | **0.767** | **0.067** | **1.035** | **0.054** | **17.822** | **0.642** | **0.690** | **0.072** |
| 106 | Ser | 1.376 | 0.036 | 14.850 | 0.403 | 0.683 | 0.040 | 1.023 | 0.037 | 16.532 | 0.338 | 0.865 | 0.061 |
| 107 | Pro | - | - | - | - | - | - | - | - | - | - | - | - |
| 108 | Val | 1.452 | 0.031 | 16.078 | 0.416 | 0.769 | 0.040 | 1.098 | 0.033 | 17.099 | 0.340 | 0.783 | 0.047 |
| 109 | Thr | o.l. | o.l. | 16.959 | 1.397 | o.l. | o.l. | o.l. | o.l. | 18.449 | 1.397 | o.l. | o.l. |
| 110 | Glu | 1.350 | 0.023 | 17.214 | 0.306 | 0.746 | 0.031 | 1.035 | 0.026 | 19.213 | 0.292 | 0.875 | 0.043 |
| 111 | Lys | 1.363 | 0.023 | 15.028 | 0.300 | 0.856 | 0.035 | 1.050 | 0.023 | 16.327 | 0.234 | 0.856 | 0.035 |
| 112 | His | 1.270 | 0.020 | 14.657 | 0.261 | 0.822 | 0.029 | 1.026 | 0.020 | 16.299 | 0.212 | 0.804 | 0.031 |
| 113 | Leu | o.l. | o.l. | o.l. | o.l. | o.l. | o.l. | o.l. | o.l. | o.l. | o.l. | o.l. | o.l. |
| 114 | Thr | 1.447 | 0.051 | 15.412 | 0.476 | 0.715 | 0.038 | 1.034 | 0.037 | 15.787 | 0.403 | 0.789 | 0.047 |
| 115 | Asp | 1.441 | 0.032 | o.l. | o.l. | o.l. | o.l. | 1.081 | 0.022 | o.l. | o.l. | o.l. | o.l. |
| 116 | Gly | 1.412 | 0.023 | 15.452 | 0.256 | 0.858 | 0.027 | 1.114 | 0.024 | 16.171 | 0.227 | 0.824 | 0.034 |
| 117 | Met | o.l. | o.l. | o.l. | o.l. | o.l. | o.l. | o.l. | o.l. | o.l. | o.l. | o.l. | o.l. |
| 118 | Thr | 1.372 | 0.031 | 14.202 | 0.378 | 0.766 | 0.035 | 1.064 | 0.031 | 15.434 | 0.338 | 0.794 | 0.040 |
| 119 | Val | 1.340 | 0.029 | 14.330 | 0.318 | 0.686 | 0.035 | 1.024 | 0.031 | 17.245 | 0.344 | 0.834 | 0.047 |
| 120 | Arg | 1.356 | 0.028 | 17.776 | 0.475 | 0.855 | 0.036 | 1.112 | 0.029 | 17.967 | 0.344 | 0.772 | 0.036 |
| 121 | Glu | 1.316 | 0.030 | 17.026 | 0.411 | 0.917 | 0.041 | 1.032 | 0.029 | 18.295 | 0.384 | 0.842 | 0.046 |
| 122 | Leu | 1.334 | 0.034 | 16.602 | 0.454 | 0.786 | 0.042 | 1.120 | 0.038 | 17.862 | 0.407 | 0.875 | 0.059 |
| 123 | Cys | o.l. | o.l. | o.l. | o.l. | o.l. | o.l. | o.l. | o.l. | o.l. | o.l. | o.l. | o.l. |
| 124 | Ser | 1.340 | 0.041 | o.l. | o.l. | 0.659 | 0.049 | 0.975 | 0.050 | o.l. | o.l. | 0.843 | 0.078 |
| 125 | Ala | 1.385 | 0.044 | 21.302 | 0.929 | 0.851 | 0.059 | 1.104 | 0.054 | 23.497 | 0.755 | 0.764 | 0.059 |
| 126 | Ala | 1.456 | 0.038 | 17.347 | 0.540 | 0.848 | 0.048 | 1.134 | 0.038 | 19.496 | 0.501 | 0.903 | 0.069 |
| 127 | Ile | 1.434 | 0.072 | 18.938 | 1.176 | 0.860 | 0.082 | 1.107 | 0.085 | 17.586 | 1.230 | 0.801 | 0.094 |
| 128 | Thr | o.l. | o.l. | o.l. | o.l. | o.l. | o.l. | o.l. | o.l. | o.l. | o.l. | o.l. | o.l. |
| 129 | Met | 1.351 | 0.064 | 15.786 | 0.876 | 0.772 | 0.080 | 1.074 | 0.069 | 18.641 | 0.851 | 0.733 | 0.078 |
| **130** | **Ser** | **o.l.** | **o.l.** | **o.l.** | **o.l.** | **o.l.** | **o.l.** | **o.l.** | **o.l.** | **o.l.** | **o.l.** | **o.l.** | **o.l.** |
| 131 | Asp | n.o. | n.o. | n.o. | n.o. | n.o. | n.o. | n.o. | n.o. | n.o. | n.o. | n.o. | n.o. |
| 132 | Asn | n.o. | n.o. | n.o. | n.o. | n.o. | n.o. | n.o. | n.o. | n.o. | n.o. | n.o. | n.o. |
| 133 | Thr | 1.399 | 0.030 | 15.771 | 0.401 | 0.899 | 0.039 | 1.142 | 0.036 | 17.267 | 0.364 | 0.850 | 0.051 |
| 134 | Ala | 1.371 | 0.033 | 15.776 | 0.409 | 0.793 | 0.042 | 1.086 | 0.040 | 16.842 | 0.416 | 0.817 | 0.052 |
| 135 | Ala | 1.308 | 0.047 | 16.071 | 0.581 | 0.911 | 0.065 | 1.095 | 0.053 | 16.208 | 0.510 | 0.709 | 0.060 |
| 136 | Asn | o.l. | o.l. | o.l. | o.l. | o.l. | o.l. | o.l. | o.l. | o.l. | o.l. | o.l. | o.l. |
| 137 | Leu | 1.477 | 0.045 | 16.794 | 0.578 | 0.830 | 0.051 | 1.092 | 0.049 | 18.121 | 0.571 | 0.769 | 0.057 |
| 138 | Leu | 1.399 | 0.032 | 16.266 | 0.398 | 0.748 | 0.035 | 1.071 | 0.034 | 18.496 | 0.378 | 0.772 | 0.043 |
| 139 | Leu | o.l. | o.l. | o.l. | o.l. | o.l. | o.l. | o.l. | o.l. | o.l. | o.l. | o.l. | o.l. |
| 140 | Thr | 1.422 | 0.024 | 14.778 | 0.293 | 0.795 | 0.032 | 1.033 | 0.024 | 15.328 | 0.256 | 0.806 | 0.038 |
| 141 | Thr | 1.415 | 0.044 | 17.030 | 0.769 | 0.850 | 0.052 | 1.185 | 0.053 | 17.851 | 0.566 | 0.933 | 0.080 |
| 142 | Ile | 1.416 | 0.030 | 14.147 | 0.372 | 0.820 | 0.037 | 1.122 | 0.034 | 15.313 | 0.349 | 0.737 | 0.041 |
| 143 | Gly | 1.383 | 0.024 | 14.362 | 0.270 | 0.849 | 0.034 | 1.082 | 0.029 | 15.036 | 0.268 | 0.859 | 0.039 |
| 144 | Gly | 1.424 | 0.020 | 11.571 | 0.171 | 0.662 | 0.025 | 1.130 | 0.027 | 12.534 | 0.174 | 0.711 | 0.030 |
| 145 | Pro | - | - | - | - | - | - | - | - | - | - | - | - |
| 146 | Lys | o.l. | o.l. | o.l. | o.l. | o.l. | o.l. | o.l. | o.l. | o.l. | o.l. | o.l. | o.l. |
| 147 | Glu | 1.366 | 0.020 | 15.142 | 0.196 | 0.823 | 0.027 | 1.090 | 0.019 | 16.520 | 0.190 | 0.889 | 0.031 |
| 148 | Leu | 1.385 | 0.030 | 15.686 | 0.296 | 0.766 | 0.035 | 1.119 | 0.025 | 16.944 | 0.218 | 0.824 | 0.036 |
| 149 | Thr | o.l. | o.l. | o.l. | o.l. | o.l. | o.l. | o.l. | o.l. | o.l. | o.l. | o.l. | o.l. |
| 150 | Asp | 1.371 | 0.019 | 16.119 | 0.231 | 0.760 | 0.024 | 1.090 | 0.019 | 17.610 | 0.186 | 0.864 | 0.032 |
| 151 | Phe | 1.414 | 0.019 | 15.673 | 0.224 | 0.879 | 0.027 | 1.107 | 0.021 | 16.432 | 0.206 | 0.823 | 0.031 |
| 152 | Leu | o.l. | o.l. | o.l. | o.l. | o.l. | o.l. | o.l. | o.l. | o.l. | o.l. | o.l. | o.l. |
| 153 | Arg | 1.381 | 0.024 | 16.384 | 0.363 | o.l. | o.l. | 1.056 | 0.024 | 17.990 | 0.272 | o.l. | o.l. |
| 154 | Gln | 1.366 | 0.018 | 15.436 | 0.221 | 0.759 | 0.024 | 1.033 | 0.019 | 17.395 | 0.198 | 0.781 | 0.029 |
| 155 | Ile | 1.314 | 0.021 | 14.091 | 0.268 | 0.775 | 0.030 | 1.047 | 0.023 | 15.537 | 0.237 | 0.805 | 0.036 |
| 156 | Gly | 1.299 | 0.023 | 15.984 | 0.313 | 0.795 | 0.028 | 1.038 | 0.027 | 17.217 | 0.304 | 0.838 | 0.038 |
| 157 | Asp | 1.273 | 0.013 | 15.794 | 0.222 | 0.748 | 0.018 | 1.017 | 0.016 | 17.828 | 0.179 | 0.780 | 0.022 |
| 158 | Lys | o.l. | o.l. | o.l. | o.l. | o.l. | o.l. | o.l. | o.l. | o.l. | o.l. | o.l. | o.l. |
| 159 | Glu | 1.397 | 0.022 | 14.969 | 0.291 | 0.778 | 0.027 | 1.096 | 0.025 | 16.111 | 0.219 | 0.799 | 0.035 |
| 160 | Thr | 1.297 | 0.032 | 14.071 | 0.411 | 0.802 | 0.040 | 1.055 | 0.036 | 15.993 | 0.368 | 0.883 | 0.059 |
| 161 | Arg | 1.471 | 0.041 | 17.589 | 0.531 | 0.805 | 0.043 | 1.136 | 0.044 | 19.599 | 0.561 | 0.819 | 0.057 |
| 162 | Leu | 1.389 | 0.025 | 16.362 | 0.356 | 0.849 | 0.029 | 0.990 | 0.031 | 18.179 | 0.299 | 0.846 | 0.040 |
| 163 | Asp | 1.198 | 0.081 | 16.900 | 1.105 | 0.781 | 0.067 | 0.931 | 0.093 | 17.590 | 1.233 | 0.821 | 0.114 |
| 164 | Arg | 1.206 | 0.063 | 16.400 | 0.905 | 0.768 | 0.067 | 0.895 | 0.070 | 16.510 | 0.842 | 0.747 | 0.074 |
| 165 | Ile | 1.228 | 0.055 | 19.746 | 0.961 | 0.815 | 0.054 | 0.939 | 0.050 | 19.479 | 0.835 | 0.810 | 0.061 |
| **166** | **Glu** | **o.l.** | **o.l.** | **o.l.** | **o.l.** | **o.l.** | **o.l.** | **o.l.** | **o.l.** | **o.l.** | **o.l.** | **o.l.** | **o.l.** |
| 167 | Pro | - | - | - | - | - | - | - | - | - | - | - | - |
| 168 | Asp | o.l. | o.l. | o.l. | o.l. | o.l. | o.l. | o.l. | o.l. | o.l. | o.l. | o.l. | o.l. |
| 169 | Leu | o.l. | o.l. | 14.888 | 1.133 | o.l. | o.l. | o.l. | o.l. | 18.213 | 1.1744 | o.l. | o.l. |
| 170 | Asn | o.l. | o.l. | o.l. | o.l. | o.l. | o.l. | o.l. | o.l. | o.l. | o.l. | o.l. | o.l. |
| 171 | Glu | 1.333 | 0.063 | 17.996 | 0.732 | 0.960 | 0.077 | 1.064 | 0.068 | 19.060 | 0.879 | 0.965 | 0.099 |

**Table S1:** cTEM-17m $^{15}$N spin relaxation data (continued)

| Residue | | 500 MHz | | | | | | 600 MHz | | | | | |
|---|---|---|---|---|---|---|---|---|---|---|---|---|---|
| # | aa | $R_1$ ($s^{-1}$) | $\Delta R_1$ ($s^{-1}$) | $R_2$ ($s^{-1}$) | $\Delta R_2$ ($s^{-1}$) | $NOE$ | $\Delta NOE$ | $R_1$ ($s^{-1}$) | $\Delta R_1$ ($s^{-1}$) | $R_2$ ($s^{-1}$) | $\Delta R_2$ ($s^{-1}$) | $NOE$ | $\Delta NOE$ |
| 172 | Gly | o.l. | o.l. | o.l. | o.l. | o.l. | o.l. | o.l. | o.l. | o.l. | o.l. | o.l. | o.l. |
| 173 | Lys | n.o. | n.o. | n.o. | n.o. | n.o. | n.o. | n.o. | n.o. | n.o. | n.o. | n.o. | n.o. |
| 174 | Leu | o.l. | o.l. | o.l. | o.l. | o.l. | o.l. | o.l. | o.l. | o.l. | o.l. | o.l. | o.l. |
| 175 | Gly | o.l. | o.l. | 16.184 | 1.889 | o.l. | o.l. | o.l. | o.l. | 20.645 | 1.749 | o.l. | o.l. |
| 176 | Asp | 1.372 | 0.021 | 14.614 | 0.230 | 0.812 | 0.027 | 1.057 | 0.020 | 16.044 | 0.217 | 0.830 | 0.034 |
| 177 | Leu | 1.393 | 0.038 | 15.564 | 0.438 | 0.727 | 0.039 | 1.177 | 0.044 | 17.328 | 0.474 | 0.757 | 0.050 |
| 178 | Arg | 1.473 | 0.029 | 16.575 | 0.312 | 0.768 | 0.026 | 1.155 | 0.027 | 18.357 | 0.289 | 0.807 | 0.036 |
| 179 | Asp | 1.388 | 0.087 | 18.814 | 1.510 | 0.770 | 0.082 | 1.066 | 0.079 | 21.677 | 1.247 | 0.767 | 0.096 |
| 180 | Thr | 1.346 | 0.043 | 17.886 | 0.608 | 0.779 | 0.042 | 1.068 | 0.039 | 19.597 | 0.577 | 0.777 | 0.048 |
| 181 | Thr | o.l. | o.l. | o.l. | o.l. | o.l. | o.l. | o.l. | o.l. | o.l. | o.l. | o.l. | o.l. |
| 182 | Thr | 1.339 | 0.020 | o.l. | o.l. | 0.837 | 0.028 | 1.042 | 0.025 | o.l. | o.l. | 0.902 | 0.038 |
| 183 | Pro | - | - | - | - | - | - | - | - | - | - | - | - |
| 184 | Lys | 1.465 | 0.042 | 14.669 | 0.476 | 0.769 | 0.044 | 1.113 | 0.046 | 15.872 | 0.453 | 0.770 | 0.058 |
| 185 | Ala | 1.397 | 0.028 | 16.212 | 0.345 | 0.804 | 0.033 | 1.105 | 0.027 | 17.074 | 0.283 | 0.843 | 0.042 |
| 186 | Ile | 1.408 | 0.027 | 16.111 | 0.333 | 0.798 | 0.033 | 1.085 | 0.027 | 16.804 | 0.268 | 0.782 | 0.035 |
| 187 | Ala | o.l. | o.l. | o.l. | o.l. | o.l. | o.l. | o.l. | o.l. | o.l. | o.l. | o.l. | o.l. |
| 188 | Ser | 1.447 | 0.023 | 14.665 | 0.253 | 0.794 | 0.028 | 1.056 | 0.022 | 15.275 | 0.206 | 0.870 | 0.036 |
| 189 | Thr | 1.384 | 0.022 | 16.256 | 0.316 | 0.775 | 0.030 | 1.058 | 0.026 | 17.045 | 0.290 | 0.793 | 0.038 |
| 190 | Leu | 1.425 | 0.027 | 16.350 | 0.329 | 0.820 | 0.034 | 1.095 | 0.028 | 18.161 | 0.333 | 0.797 | 0.039 |
| 191 | Arg | 1.419 | 0.027 | 15.578 | 0.336 | 0.818 | 0.035 | 1.092 | 0.028 | 16.329 | 0.320 | 0.747 | 0.036 |
| 192 | Lys | 1.419 | 0.021 | 15.172 | 0.245 | 0.754 | 0.027 | 1.149 | 0.023 | 16.737 | 0.229 | 0.852 | 0.036 |
| 193 | Leu | 1.402 | 0.026 | 14.842 | 0.335 | 0.812 | 0.032 | 1.053 | 0.029 | 16.398 | 0.285 | 0.805 | 0.044 |
| 194 | Leu | o.l. | o.l. | o.l. | o.l. | 0.879 | 0.094 | o.l. | o.l. | o.l. | o.l. | 0.935 | 0.080 |
| 195 | Thr | 1.448 | 0.033 | 14.614 | 0.317 | 0.793 | 0.033 | 1.149 | 0.031 | 15.701 | 0.318 | 0.803 | 0.040 |
| 196 | Gly | 1.639 | 0.026 | 13.737 | 0.261 | 0.785 | 0.025 | 1.309 | 0.032 | 14.475 | 0.224 | 0.811 | 0.035 |
| 197 | Glu | 1.384 | 0.032 | 14.050 | 0.287 | 0.705 | 0.032 | 1.052 | 0.025 | 15.549 | 0.259 | 0.734 | 0.034 |
| 198 | Leu | 1.219 | 0.015 | 13.104 | 0.156 | 0.557 | 0.022 | 0.966 | 0.015 | 14.489 | 0.147 | 0.618 | 0.023 |
| 199 | Leu | o.l. | o.l. | o.l. | o.l. | o.l. | o.l. | o.l. | o.l. | o.l. | o.l. | o.l. | o.l. |
| 200 | Thr | 1.330 | 0.033 | 15.071 | 0.418 | 0.835 | 0.038 | 1.034 | 0.032 | 16.316 | 0.352 | 0.838 | 0.044 |
| 201 | Leu | 1.445 | 0.019 | 15.114 | 0.222 | 0.824 | 0.023 | 1.142 | 0.020 | 15.679 | 0.186 | 0.832 | 0.028 |
| 202 | Ala | 1.404 | 0.017 | 15.626 | 0.197 | 0.767 | 0.022 | 1.134 | 0.016 | 16.565 | 0.161 | 0.819 | 0.026 |
| 203 | Ser | 1.411 | 0.017 | 15.204 | 0.204 | 0.806 | 0.021 | 1.049 | 0.017 | 16.119 | 0.174 | 0.839 | 0.026 |
| 204 | Arg | 1.432 | 0.021 | 15.986 | 0.251 | 0.798 | 0.026 | 1.096 | 0.022 | 17.217 | 0.210 | 0.816 | 0.033 |
| 205 | Gln | 1.423 | 0.030 | 16.493 | 0.406 | 0.859 | 0.043 | 1.045 | 0.032 | 17.678 | 0.359 | 0.849 | 0.050 |
| 206 | Gln | 1.380 | 0.021 | 15.372 | 0.264 | 0.856 | 0.029 | 1.066 | 0.021 | 17.042 | 0.232 | 0.821 | 0.030 |
| 207 | Leu | 1.234 | 0.020 | 9.322 | 0.155 | 0.478 | 0.030 | 1.020 | 0.024 | 12.457 | 0.179 | 0.756 | 0.037 |
| 208 | Ile | 1.424 | 0.039 | 16.899 | 0.609 | 0.820 | 0.049 | 1.049 | 0.043 | 18.281 | 0.477 | 0.946 | 0.072 |
| 209 | Asp | 1.408 | 0.028 | 16.587 | 0.383 | 0.805 | 0.039 | 1.108 | 0.035 | 17.818 | 0.384 | 0.819 | 0.049 |
| 210 | Trp | 1.411 | 0.022 | 15.718 | 0.249 | 0.740 | 0.027 | 1.110 | 0.025 | 17.438 | 0.265 | 0.807 | 0.036 |
| 211 | Met | 1.479 | 0.039 | 15.394 | 0.505 | 0.897 | 0.045 | 1.136 | 0.042 | 17.786 | 0.397 | 0.793 | 0.053 |
| 212 | Glu | o.l. | o.l. | o.l. | o.l. | o.l. | o.l. | o.l. | o.l. | o.l. | o.l. | o.l. | o.l. |
| 213 | Ala | 1.506 | 0.075 | 20.419 | 1.185 | 0.790 | 0.067 | 1.019 | 0.066 | 24.429 | 1.208 | 0.811 | 0.076 |
| 214 | Asp | n.o. | n.o. | n.o. | n.o. | n.o. | n.o. | n.o. | n.o. | n.o. | n.o. | n.o. | n.o. |
| 215 | Lys | o.l. | o.l. | o.l. | o.l. | o.l. | o.l. | o.l. | o.l. | o.l. | o.l. | o.l. | o.l. |
| 216 | Val | n.o. | n.o. | n.o. | n.o. | n.o. | n.o. | n.o. | n.o. | n.o. | n.o. | n.o. | n.o. |
| 217 | Ala | n.o. | n.o. | n.o. | n.o. | n.o. | n.o. | n.o. | n.o. | n.o. | n.o. | n.o. | n.o. |
| 218 | Gly | n.o. | n.o. | n.o. | n.o. | n.o. | n.o. | n.o. | n.o. | n.o. | n.o. | n.o. | n.o. |
| 219 | Pro | - | - | - | - | - | - | - | - | - | - | - | - |
| 220 | Leu | n.o. | n.o. | n.o. | n.o. | n.o. | n.o. | n.o. | n.o. | n.o. | n.o. | n.o. | n.o. |
| 221 | Leu | n.o. | n.o. | n.o. | n.o. | n.o. | n.o. | n.o. | n.o. | n.o. | n.o. | n.o. | n.o. |
| 222 | Arg | o.l. | o.l. | o.l. | o.l. | o.l. | o.l. | o.l. | o.l. | o.l. | o.l. | o.l. | o.l. |
| 223 | Ser | 1.476 | 0.066 | o.l. | o.l. | 0.806 | 0.056 | 1.179 | 0.069 | o.l. | o.l. | 0.951 | 0.086 |
| 224 | Ala | 1.338 | 0.033 | 14.678 | 0.381 | 0.801 | 0.048 | 1.126 | 0.038 | 16.285 | 0.362 | 0.814 | 0.058 |
| 225 | Leu | 1.207 | 0.039 | 16.031 | 0.583 | 0.739 | 0.047 | 0.957 | 0.048 | 17.056 | 0.570 | 0.821 | 0.063 |
| 226 | Pro | - | - | - | - | - | - | - | - | - | - | - | - |
| 227 | Ala | 1.272 | 0.014 | 14.767 | 0.175 | 0.804 | 0.020 | 1.024 | 0.016 | 16.238 | 0.157 | 0.839 | 0.026 |
| 228 | Gly | 1.241 | 0.045 | 14.499 | 0.562 | 0.805 | 0.048 | 0.968 | 0.036 | 15.424 | 0.436 | 0.884 | 0.049 |
| 229 | Trp | 1.411 | 0.022 | 15.713 | 0.230 | 0.840 | 0.028 | 1.080 | 0.026 | 16.921 | 0.225 | 0.843 | 0.036 |
| 230 | Phe | 1.369 | 0.035 | 17.872 | 0.609 | 0.758 | 0.041 | 1.021 | 0.041 | 19.065 | 0.466 | 0.765 | 0.045 |
| 231 | Ile | 1.392 | 0.066 | 17.822 | 0.901 | 0.772 | 0.077 | 0.957 | 0.072 | 23.173 | 1.334 | 0.705 | 0.077 |
| 232 | Ala | 1.309 | 0.079 | 17.604 | 1.084 | o.l. | o.l. | 1.062 | 0.099 | 21.205 | 1.222 | o.l. | o.l. |
| 233 | Asp | 1.412 | 0.074 | 16.773 | 0.744 | 0.677 | 0.069 | 1.043 | 0.073 | 18.276 | 0.870 | 0.772 | 0.090 |
| **234** | **Lys** | **n.o.** | **n.o.** | **n.o.** | **n.o.** | **n.o.** | **n.o.** | **n.o.** | **n.o.** | **n.o.** | **n.o.** | **n.o.** | **n.o.** |
| 235 | Ser | n.o. | n.o. | n.o. | n.o. | n.o. | n.o. | n.o. | n.o. | n.o. | n.o. | n.o. | n.o. |
| 236 | Gly | n.o. | n.o. | n.o. | n.o. | n.o. | n.o. | n.o. | n.o. | n.o. | n.o. | n.o. | n.o. |
| 237 | Ala | n.o. | n.o. | n.o. | n.o. | n.o. | n.o. | n.o. | n.o. | n.o. | n.o. | n.o. | n.o. |
| 238 | Gly | n.o. | n.o. | n.o. | n.o. | n.o. | n.o. | n.o. | n.o. | n.o. | n.o. | n.o. | n.o. |
| 240 | Glu | n.o. | n.o. | n.o. | n.o. | n.o. | n.o. | n.o. | n.o. | n.o. | n.o. | n.o. | n.o. |
| 241 | Arg | n.o. | n.o. | n.o. | n.o. | n.o. | n.o. | n.o. | n.o. | n.o. | n.o. | n.o. | n.o. |
| 242 | Gly | n.o. | n.o. | n.o. | n.o. | n.o. | n.o. | n.o. | n.o. | n.o. | n.o. | n.o. | n.o. |
| 243 | Ser | n.o. | n.o. | n.o. | n.o. | n.o. | n.o. | n.o. | n.o. | n.o. | n.o. | n.o. | n.o. |
| 244 | Arg | n.o. | n.o. | n.o. | n.o. | n.o. | n.o. | n.o. | n.o. | n.o. | n.o. | n.o. | n.o. |
| 245 | Gly | o.l. | o.l. | o.l. | o.l. | o.l. | o.l. | o.l. | o.l. | o.l. | o.l. | o.l. | o.l. |

**Table S1:** cTEM-17m $^{15}$N spin relaxation data (continued)

| Residue | | 500 MHz | | | | | | 600 MHz | | | | | |
|---|---|---|---|---|---|---|---|---|---|---|---|---|---|
| # | aa | $R_1$ (s$^{-1}$) | $\Delta R_1$ (s$^{-1}$) | $R_2$ (s$^{-1}$) | $\Delta R_2$ (s$^{-1}$) | $NOE$ | $\Delta NOE$ | $R_1$ (s$^{-1}$) | $\Delta R_1$ (s$^{-1}$) | $R_2$ (s$^{-1}$) | $\Delta R_2$ (s$^{-1}$) | $NOE$ | $\Delta NOE$ |
| 246 | Ile | 1.578 | 0.107 | o.l. | o.l. | 0.782 | 0.095 | 1.096 | 0.102 | o.l. | o.l. | 0.925 | 0.114 |
| 247 | Ile | o.l. | o.l. | o.l. | o.l. | o.l. | o.l. | o.l. | o.l. | o.l. | o.l. | o.l. | o.l. |
| 248 | Ala | 1.347 | 0.035 | 16.883 | 0.433 | 0.794 | 0.048 | 1.060 | 0.041 | 17.464 | 0.476 | 0.858 | 0.064 |
| 249 | Ala | 1.365 | 0.039 | 16.336 | 0.490 | 0.725 | 0.044 | 1.100 | 0.043 | 18.590 | 0.534 | 0.929 | 0.060 |
| 250 | Leu | 1.346 | 0.043 | 16.524 | 0.654 | 0.789 | 0.056 | 1.119 | 0.059 | 18.152 | 0.684 | 0.759 | 0.065 |
| 251 | Gly | 1.402 | 0.036 | 15.409 | 0.445 | 0.796 | 0.041 | 1.037 | 0.045 | 18.317 | 0.515 | 0.833 | 0.053 |
| 252 | Pro | - | - | - | - | - | - | - | - | - | - | - | - |
| 254 | Asp | 1.304 | 0.031 | 17.775 | 0.486 | 0.858 | 0.042 | 1.051 | 0.040 | 20.018 | 0.547 | 0.858 | 0.056 |
| 255 | Gly | 1.388 | 0.046 | 21.571 | 0.807 | 0.727 | 0.049 | 1.128 | 0.067 | 26.365 | 1.115 | 0.836 | 0.076 |
| 256 | Lys | 1.271 | 0.013 | 15.776 | 0.192 | 0.805 | 0.022 | 1.016 | 0.015 | 18.022 | 0.172 | 0.822 | 0.025 |
| 257 | Pro | - | - | - | - | - | - | - | - | - | - | - | - |
| 258 | Ser | 1.423 | 0.084 | 17.150 | 1.352 | 0.796 | 0.097 | 1.213 | 0.111 | 16.859 | 1.232 | 0.769 | 0.105 |
| 259 | Arg | 1.346 | 0.039 | o.l. | o.l. | 0.716 | 0.043 | 1.088 | 0.039 | o.l. | o.l. | 0.810 | 0.052 |
| 260 | Ile | 1.303 | 0.037 | 16.415 | 0.523 | 0.766 | 0.049 | 1.043 | 0.043 | 16.759 | 0.517 | 0.801 | 0.063 |
| 261 | Val | 1.342 | 0.048 | 15.675 | 0.632 | 0.701 | 0.057 | 1.064 | 0.058 | 17.241 | 0.631 | 0.853 | 0.081 |
| 262 | Val | 1.227 | 0.048 | 15.920 | 0.646 | 0.753 | 0.063 | 1.099 | 0.063 | 16.925 | 0.687 | 0.808 | 0.084 |
| 263 | Ile | o.l. | o.l. | o.l. | o.l. | o.l. | o.l. | o.l. | o.l. | o.l. | o.l. | o.l. | o.l. |
| 264 | Tyr | 1.438 | 0.066 | 18.546 | 0.974 | 0.842 | 0.081 | 1.095 | 0.076 | 21.792 | 1.070 | 0.838 | 0.091 |
| 265 | Thr | o.l. | o.l. | 24.727 | 2.490 | o.l. | o.l. | o.l. | o.l. | 26.470 | 2.520 | o.l. | o.l. |
| 266 | Thr | n.o. | n.o. | n.o. | n.o. | n.o. | n.o. | n.o. | n.o. | n.o. | n.o. | n.o. | n.o. |
| 267 | Gly | n.o. | n.o. | n.o. | n.o. | n.o. | n.o. | n.o. | n.o. | n.o. | n.o. | n.o. | n.o. |
| 268 | Ser | 1.561 | 0.094 | 17.110 | 1.057 | 0.809 | 0.076 | 1.304 | 0.109 | 17.872 | 0.969 | 0.819 | 0.107 |
| 269 | Gln | 1.429 | 0.053 | o.l. | o.l. | 0.726 | 0.048 | 1.118 | 0.046 | o.l. | o.l. | 0.831 | 0.054 |
| 270 | Ala | o.l. | o.l. | o.l. | o.l. | o.l. | o.l. | o.l. | o.l. | o.l. | o.l. | o.l. | o.l. |
| 271 | Thr | 1.413 | 0.041 | 15.651 | 0.537 | 0.777 | 0.045 | 1.100 | 0.041 | 17.410 | 0.481 | 0.712 | 0.052 |
| 272 | Met | n.o. | n.o. | n.o. | n.o. | n.o. | n.o. | n.o. | n.o. | n.o. | n.o. | n.o. | n.o. |
| 273 | Asp | 1.534 | 0.028 | 15.732 | 0.337 | 0.818 | 0.034 | 1.086 | 0.025 | 16.659 | 0.272 | 0.828 | 0.038 |
| 274 | Glu | 1.378 | 0.023 | 15.626 | 0.281 | 0.842 | 0.029 | 1.076 | 0.024 | 17.781 | 0.258 | 0.826 | 0.034 |
| 275 | Arg | o.l. | o.l. | o.l. | o.l. | o.l. | o.l. | o.l. | o.l. | o.l. | o.l. | o.l. | o.l. |
| 276 | Asn | o.l. | o.l. | o.l. | o.l. | o.l. | o.l. | o.l. | o.l. | o.l. | o.l. | o.l. | o.l. |
| 277 | Arg | o.l. | o.l. | o.l. | o.l. | o.l. | o.l. | o.l. | o.l. | o.l. | o.l. | o.l. | o.l. |
| 278 | Gln | 1.397 | 0.060 | 15.726 | 0.802 | 0.696 | 0.068 | 1.106 | 0.071 | 17.519 | 0.819 | 0.730 | 0.077 |
| 279 | Ile | 1.434 | 0.088 | 16.955 | 1.362 | o.l. | o.l. | 1.305 | 0.114 | 21.319 | 1.645 | 0.785 | 0.103 |
| 280 | Ala | 1.388 | 0.026 | 12.989 | 0.240 | 0.625 | 0.035 | 1.157 | 0.029 | 15.914 | 0.275 | 0.670 | 0.035 |
| 281 | Glu | 1.361 | 0.031 | 16.103 | 0.422 | 0.846 | 0.040 | 1.169 | 0.049 | 16.495 | 0.423 | o.l. | o.l. |
| 282 | Ile | 1.371 | 0.044 | 15.783 | 0.539 | 0.811 | 0.055 | 1.073 | 0.047 | 19.720 | 0.667 | 0.934 | 0.085 |
| 283 | Gly | 1.561 | 0.063 | 16.222 | 0.711 | 0.876 | 0.063 | 1.067 | 0.062 | 18.221 | 0.763 | 0.740 | 0.067 |
| 284 | Ala | 1.422 | 0.038 | 16.132 | 0.460 | 0.871 | 0.050 | 1.198 | 0.045 | 18.166 | 0.526 | 0.838 | 0.058 |
| 285 | Ser | 1.446 | 0.034 | 15.123 | 0.436 | 0.804 | 0.041 | 1.074 | 0.035 | 16.166 | 0.401 | 0.876 | 0.056 |
| 286 | Leu | 1.338 | 0.030 | 14.686 | 0.308 | 0.809 | 0.041 | 1.094 | 0.034 | 16.478 | 0.399 | 0.864 | 0.053 |
| 287 | Ile | 1.394 | 0.056 | 20.556 | 1.086 | 0.693 | 0.061 | 1.174 | 0.073 | 25.224 | 1.114 | 0.890 | 0.105 |
| 288 | Lys | o.l. | o.l. | o.l. | o.l. | o.l. | o.l. | o.l. | o.l. | o.l. | o.l. | o.l. | o.l. |
| 289 | His | 1.312 | 0.035 | 14.936 | 0.399 | 0.877 | 0.045 | 1.037 | 0.039 | 17.344 | 0.392 | 0.734 | 0.050 |
| 290 | Trp | 1.358 | 0.025 | 13.326 | 0.292 | 0.788 | 0.033 | 1.038 | 0.029 | 15.097 | 0.271 | 0.756 | 0.039 |

- Values presented here are rounded to three decimals. Exact values can be obtained from the BMRB (accession number 16598).
- Important active site residues (Ser[70], Lys[73], Tyr[105], Ser[130], Glu[166], and Arg[234]) are shown in bold red while residues from the Ω loop (residues 161–179) are coloured blue.
- n-ter: N-terminus amine (not observable).
- n.o.: non-observed N-H resonances (not assigned).
- o.l.: overlapped N-H resonances.
