# Supplementary material for: Chimeric β-Lactamases: Global Conservation of Parental Function and Fast Time-Scale Dynamics with Increased Slow Motions
Source: PLoS One. 2012 Dec 21;7(12):e52283. doi: 10.1371/journal.pone.0052283 (PMC3528772; doi:10.1371/journal.pone.0052283)
Supplement: Table S2 — cTEM-17m model-free parameters. (PDF) [file pone.0052283.s009.pdf]

Table S2: cTEM-17m model-free parameters

Residue #	aa	Model	Parameters	Diffusion core ?	S^2	ΔS^2	S_f^2	ΔS_f^2	S_s^2	ΔS_s^2	τ_e ps	$\Delta\tau_e$ ps	τ_f ps	$\Delta\tau_f$ ps	τ_s ps	$\Delta\tau_s$ ps	$^{600}R_{ex}$ s^{-1}	$\Delta^{600}R_{ex}$ s^{-1}
26	His	n-ter	-	-	-	-	-	-	-	-	-	-	-	-	-	-	-	-
27	Pro	-	-	-	-	-	-	-	-	-	-	-	-	-	-	-	-	-
28	Glu	m3	S^2,R_{ex}	yes	0.840	0.017	-	-	-	-	-	-	-	-	-	-	1.295	0.300
29	Thr	m1	S^2	yes	0.946	0.014	-	-	-	-	-	-	-	-	-	-	-	-
30	Leu	m3	S^2,R_{ex}	yes	0.891	0.016	-	-	-	-	-	-	-	-	-	-	0.885	0.310
31	Val	m3	S^2,R_{ex}	yes	0.862	0.011	-	-	-	-	-	-	-	-	-	-	1.438	0.200
32	Lys	m3	S^2,R_{ex}	yes	0.894	0.020	-	-	-	-	-	-	-	-	-	-	2.830	0.390
33	Val	o.l.	-	-	-	-	-	-	-	-	-	-	-	-	-	-	-	-
34	Lys	m1	S^2	yes	0.916	0.009	-	-	-	-	-	-	-	-	-	-	-	-
35	Asp	m2	S^2,τ_e	yes	0.926	0.011	-	-	-	-	75.249	27.981	-	-	-	-	-	-
36	Ala	m3	S^2,R_{ex}	yes	0.896	0.018	-	-	-	-	-	-	-	-	-	-	1.209	0.340
37	Glu	m3	S^2,R_{ex}	yes	0.922	0.011	-	-	-	-	-	-	-	-	-	-	1.036	0.210
38	Asp	m3	S^2,R_{ex}	yes	0.907	0.013	-	-	-	-	-	-	-	-	-	-	1.050	0.260
39	Gln	m2	S^2,τ_e	yes	0.926	0.012	-	-	-	-	61.698	26.539	-	-	-	-	-	-
40	Leu	m1	S^2	yes	0.948	0.021	-	-	-	-	-	-	-	-	-	-	-	-
41	Gly	m9	R_{ex}	-	-	-	-	-	-	-	-	-	-	-	-	-	0.960	0.430
42	Ala	o.l.	-	-	-	-	-	-	-	-	-	-	-	-	-	-	-	-
43	Arg	m2	S^2,τ_e	yes	0.792	0.019	-	-	-	-	42.370	10.778	-	-	-	-	-	-
44	Val	o.l.	-	-	-	-	-	-	-	-	-	-	-	-	-	-	-	-
45	Gly	o.l.	-	-	-	-	-	-	-	-	-	-	-	-	-	-	-	-
46	Tyr	m3	S^2,R_{ex}	yes	0.867	0.020	-	-	-	-	-	-	-	-	-	-	1.279	0.400
47	Ile	m1	S^2	yes	0.940	0.013	-	-	-	-	-	-	-	-	-	-	-	-
48	Glu	m1	S^2	yes	0.890	0.020	-	-	-	-	-	-	-	-	-	-	-	-
49	Leu	m3	S^2,R_{ex}	yes	0.842	0.022	-	-	-	-	-	-	-	-	-	-	1.320	0.430
50	Asp	m3	S^2,R_{ex}	yes	0.917	0.030	-	-	-	-	-	-	-	-	-	-	2.113	0.590
51	Leu	m1	S^2	-	0.908	0.021	-	-	-	-	-	-	-	-	-	-	-	-
52	Asn	m5	S_f^2,S^2,τ_s	-	0.784	0.018	0.878	0.015	0.894	0.015	-	-	-	-	1152.505	213.664	-	-
53	Ser	m5	S_f^2,S^2,τ_s	-	0.829	0.014	0.902	0.011	0.919	0.012	-	-	-	-	1481.522	384.177	-	-
54	Gly	m3	S^2,R_{ex}	-	0.924	0.014	-	-	-	-	-	-	-	-	-	-	0.577	0.240
55	Lys	o.l.	-	-	-	-	-	-	-	-	-	-	-	-	-	-	-	-
56	Ile	m1	S^2	yes	0.856	0.008	-	-	-	-	-	-	-	-	-	-	-	-
57	Leu	m1	S^2	yes	0.908	0.026	-	-	-	-	-	-	-	-	-	-	-	-
58	Glu	m2	S^2,τ_e	yes	0.878	0.009	-	-	-	-	31.508	9.664	-	-	-	-	-	-
59	Ser	m1	S^2	yes	0.890	0.012	-	-	-	-	-	-	-	-	-	-	-	-
60	Phe	m1	S^2	yes	0.900	0.010	-	-	-	-	-	-	-	-	-	-	-	-
61	Arg	m1	S^2	-	0.928	0.014	-	-	-	-	-	-	-	-	-	-	-	-
62	Pro	-	-	-	-	-	-	-	-	-	-	-	-	-	-	-	-	-
63	Glu	m1	S^2	-	0.901	0.009	-	-	-	-	-	-	-	-	-	-	-	-
64	Glu	m1	S^2	-	0.932	0.011	-	-	-	-	-	-	-	-	-	-	-	-
65	Arg	m1	S^2	-	0.818	0.008	-	-	-	-	-	-	-	-	-	-	-	-
66	Phe	m0	-	yes	-	-	-	-	-	-	-	-	-	-	-	-	-	-
67	Pro	-	-	-	-	-	-	-	-	-	-	-	-	-	-	-	-	-
68	Met	o.l.	-	-	-	-	-	-	-	-	-	-	-	-	-	-	-	-
69	Met	o.l.	-	-	-	-	-	-	-	-	-	-	-	-	-	-	-	-
**70**	**Ser**	**n.o.**	-	-	-	-	-	-	-	-	-	-	-	-	-	-	-	-
71	Thr	o.l.	-	-	-	-	-	-	-	-	-	-	-	-	-	-	-	-
72	Phe	o.l.	-	-	-	-	-	-	-	-	-	-	-	-	-	-	-	-
**73**	**Lys**	**m9**	R_{ex}	**yes**	-	-	-	-	-	-	-	-	-	-	-	-	**1.527**	**0.520**
74	Val	o.l.	-	-	-	-	-	-	-	-	-	-	-	-	-	-	-	-
75	Leu	m1	S^2	yes	0.948	0.023	-	-	-	-	-	-	-	-	-	-	-	-
76	Leu	o.l.	-	-	-	-	-	-	-	-	-	-	-	-	-	-	-	-
77	Cys	m1	S^2	yes	0.882	0.022	-	-	-	-	-	-	-	-	-	-	-	-

Table S2: cTEM-17m model-free analysis results (continued)

#	aa	Model	Parameters	Diffusion core ?	S^2	ΔS^2	S^2_f	ΔS^2_f	S^2_s	ΔS^2_s	τ_e ps	$\Delta\tau_e$ ps	τ_f ps	$\Delta\tau_f$ ps	τ_s ps	$\Delta\tau_s$ ps	$^{600}R_{ex}$ s^{-1}	$\Delta^{600}R_{ex}$ s^{-1}
78	Gly	m1	S^2	yes	0.935	0.014	-	-	-	-	-	-	-	-	-	-	-	-
79	Ala	m1	S^2	yes	0.947	0.014	-	-	-	-	-	-	-	-	-	-	-	-
80	Val	o.l.	-	-	-	-	-	-	-	-	-	-	-	-	-	-	-	-
81	Leu	m1	S^2	yes	0.954	0.010	-	-	-	-	-	-	-	-	-	-	-	-
82	Ser	m1	S^2	yes	0.919	0.007	-	-	-	-	-	-	-	-	-	-	-	-
83	Arg	m1	S^2	yes	0.942	0.009	-	-	-	-	-	-	-	-	-	-	-	-
84	Val	m1	S^2	yes	0.891	0.008	-	-	-	-	-	-	-	-	-	-	-	-
85	Asp	o.l.	-	-	-	-	-	-	-	-	-	-	-	-	-	-	-	-
86	Ala	m1	S^2	-	0.886	0.006	-	-	-	-	-	-	-	-	-	-	-	-
87	Gly	m1	S^2	-	0.891	0.006	-	-	-	-	-	-	-	-	-	-	-	-
88	Gln	o.l.	-	-	-	-	-	-	-	-	-	-	-	-	-	-	-	-
89	Glu	m3	S^2,R_{ex}	-	0.812	0.007	-	-	-	-	-	-	-	-	-	-	0.333	0.120
90	Gln	m1	S^2	-	0.841	0.008	-	-	-	-	-	-	-	-	-	-	-	-
91	Leu	m3	S^2,R_{ex}	-	0.883	0.011	-	-	-	-	-	-	-	-	-	-	0.499	0.200
92	Gly	m1	S^2	-	0.939	0.011	-	-	-	-	-	-	-	-	-	-	-	-
93	Arg	m3	S^2,R_{ex}	-	0.916	0.010	-	-	-	-	-	-	-	-	-	-	0.792	0.180
94	Arg	o.l.	-	-	-	-	-	-	-	-	-	-	-	-	-	-	-	-
95	Ile	m1	S^2	yes	0.901	0.011	-	-	-	-	-	-	-	-	-	-	-	-
96	His	m1	S^2	-	0.859	0.007	-	-	-	-	-	-	-	-	-	-	-	-
97	Tyr	m4	S^2,τ_e,R_{ex}	-	0.805	0.010	-	-	-	-	17.454	4.759	-	-	-	-	1.745	4.750
98	Ser	m1	S^2	-	0.840	0.008	-	-	-	-	-	-	-	-	-	-	-	-
99	Gln	m3	S^2,R_{ex}	-	0.930	0.010	-	-	-	-	-	-	-	-	-	-	0.678	0.200
100	Asn	m1	S^2	-	0.959	0.012	-	-	-	-	-	-	-	-	-	-	-	-
101	Asp	m3	S^2,R_{ex}	-	0.857	0.009	-	-	-	-	-	-	-	-	-	-	0.920	0.150
102	Leu	m3	S^2,R_{ex}	-	0.876	0.009	-	-	-	-	-	-	-	-	-	-	0.553	0.170
103	Val	m3	S^2,R_{ex}	-	0.837	0.022	-	-	-	-	-	-	-	-	-	-	1.945	0.430
104	Glu	m1	S^2	-	0.916	0.010	-	-	-	-	-	-	-	-	-	-	-	-
**105**	**Tyr**	**m3**	S^2,R_{ex}	**-**	**0.891**	**0.027**	**-**	**-**	**-**	**-**	**-**	**-**	**-**	**-**	**-**	**-**	**1.223**	**0.490**
106	Ser	m1	S^2	-	0.903	0.011	-	-	-	-	-	-	-	-	-	-	-	-
107	Pro	-	-	-	-	-	-	-	-	-	-	-	-	-	-	-	-	-
108	Val	m3	S^2,R_{ex}	-	0.915	0.017	-	-	-	-	-	-	-	-	-	-	0.992	0.300
109	Thr	o.l.	-	-	-	-	-	-	-	-	-	-	-	-	-	-	-	-
110	Glu	m1	S^2	yes	0.966	0.008	-	-	-	-	-	-	-	-	-	-	-	-
111	Lys	m1	S^2	yes	0.897	0.008	-	-	-	-	-	-	-	-	-	-	-	-
112	His	m3	S^2,R_{ex}	-	0.822	0.010	-	-	-	-	-	-	-	-	-	-	1.386	0.180
113	Leu	o.l.	-	-	-	-	-	-	-	-	-	-	-	-	-	-	-	-
114	Thr	m2	S^2,τ_e	yes	0.887	0.013	-	-	-	-	35.806	13.162	-	-	-	-	-	-
115	Asp	o.l.	-	-	-	-	-	-	-	-	-	-	-	-	-	-	-	-
116	Gly	m1	S^2	yes	0.921	0.008	-	-	-	-	-	-	-	-	-	-	-	-
117	Met	o.l.	-	-	-	-	-	-	-	-	-	-	-	-	-	-	-	-
118	Thr	m1	S^2	yes	0.879	0.011	-	-	-	-	-	-	-	-	-	-	-	-
119	Val	m2	S^2,τ_e	yes	0.876	0.010	-	-	-	-	36.186	10.864	-	-	-	-	-	-
120	Arg	m1	S^2	yes	0.962	0.011	-	-	-	-	-	-	-	-	-	-	-	-
121	Glu	m1	S^2	yes	0.940	0.011	-	-	-	-	-	-	-	-	-	-	-	-
122	Leu	m1	S^2	yes	0.943	0.013	-	-	-	-	-	-	-	-	-	-	-	-
123	Cys	o.l.	-	-	-	-	-	-	-	-	-	-	-	-	-	-	-	-
124	Ser	v	-	-	-	-	-	-	-	-	-	-	-	-	-	-	-	-
125	Ala	m9	R_{ex}	yes	-	-	-	-	-	-	-	-	-	-	-	-	2.887	0.420
126	Ala	m0	-	yes	-	-	-	-	-	-	-	-	-	-	-	-	-	-
127	Ile	m0	-	yes	-	-	-	-	-	-	-	-	-	-	-	-	-	-
128	Thr	o.l.	-	-	-	-	-	-	-	-	-	-	-	-	-	-	-	-
129	Met	m1	S^2	-	0.944	0.024	-	-	-	-	-	-	-	-	-	-	-	-

Table S2: cTEM-17m model-free analysis results (continued)

#	aa	Model	Parameters	Diffusion core ?	S^2	ΔS^2	S^2_f	ΔS^2_f	S^2_s	ΔS^2_s	τ_e ps	$\Delta\tau_e$ ps	τ_f ps	$\Delta\tau_f$ ps	τ_s ps	$\Delta\tau_s$ ps	$^{600}R_{ex}$ s^{-1}	$\Delta^{600}R_{ex}$ s^{-1}
**130**	**Ser**	**o.l.**	**-**	**-**	**-**	**-**	**-**	**-**	**-**	**-**	**-**	**-**	**-**	**-**	**-**	**-**	**-**	**-**
131	Asp	n.o.	-	-	-	-	-	-	-	-	-	-	-	-	-	-	-	-
132	Asn	n.o.	-	-	-	-	-	-	-	-	-	-	-	-	-	-	-	-
133	Thr	m3	S^2,R_{ex}	yes	0.913	0.016	-	-	-	-	-	-	-	-	-	-	0.833	0.290
134	Ala	m1	S^2	yes	0.923	0.012	-	-	-	-	-	-	-	-	-	-	-	-
135	Ala	m1	S^2	yes	0.904	0.018	-	-	-	-	-	-	-	-	-	-	-	-
136	Asn	o.l.	-	-	-	-	-	-	-	-	-	-	-	-	-	-	-	-
137	Leu	m0	-	yes	-	-	-	-	-	-	-	-	-	-	-	-	-	-
138	Leu	m1	S^2	yes	0.961	0.012	-	-	-	-	-	-	-	-	-	-	-	-
139	Leu	o.l.	-	-	-	-	-	-	-	-	-	-	-	-	-	-	-	-
140	Thr	m1	S^2	yes	0.888	0.008	-	-	-	-	-	-	-	-	-	-	-	-
141	Thr	m0	-	yes	-	-	-	-	-	-	-	-	-	-	-	-	-	-
142	Ile	m5	S^2_f,S^2,τ_s	-	0.835	0.018	0.884	0.013	0.945	0.014	-	-	-	-	2262.029	1781.916	-	-
143	Gly	m1	S^2	-	0.872	0.009	-	-	-	-	-	-	-	-	-	-	-	-
144	Gly	m5	S^2_f,S^2,τ_s	-	0.710	0.009	0.820	0.008	0.866	0.009	-	-	-	-	1515.275	169.289	-	-
145	Pro	-	-	-	-	-	-	-	-	-	-	-	-	-	-	-	-	-
146	Lys	o.l.	-	-	-	-	-	-	-	-	-	-	-	-	-	-	-	-
147	Glu	m3	S^2,R_{ex}	yes	0.889	0.010	-	-	-	-	-	-	-	-	-	-	0.583	0.180
148	Leu	m3	S^2,R_{ex}	yes	0.913	0.013	-	-	-	-	-	-	-	-	-	-	0.563	0.220
149	Thr	o.l.	-	-	-	-	-	-	-	-	-	-	-	-	-	-	-	-
150	Asp	m1	S^2	yes	0.942	0.006	-	-	-	-	-	-	-	-	-	-	-	-
151	Phe	m1	S^2	yes	0.926	0.007	-	-	-	-	-	-	-	-	-	-	-	-
152	Leu	o.l.	-	-	-	-	-	-	-	-	-	-	-	-	-	-	-	-
153	Arg	o.l.	-	-	-	-	-	-	-	-	-	-	-	-	-	-	-	-
154	Gln	m3	S^2,R_{ex}	-	0.889	0.009	-	-	-	-	-	-	-	-	-	-	0.809	0.180
155	Ile	m1	S^2	-	0.864	0.008	-	-	-	-	-	-	-	-	-	-	-	-
156	Gly	m1	S^2	-	0.909	0.009	-	-	-	-	-	-	-	-	-	-	-	-
157	Asp	m2	S^2,τ_e	-	0.899	0.005	-	-	-	-	30.512	6.531	-	-	-	-	-	-
158	Lys	o.l.	-	-	-	-	-	-	-	-	-	-	-	-	-	-	-	-
159	Glu	m1	S^2	-	0.909	0.008	-	-	-	-	-	-	-	-	-	-	-	-
160	Thr	m3	S^2,R_{ex}	-	0.840	0.017	-	-	-	-	-	-	-	-	-	-	0.731	0.300
161	Arg	m9	R_{ex}	-	-	-	-	-	-	-	-	-	-	-	-	-	0.625	0.300
162	Leu	m3	S^2,R_{ex}	-	0.914	0.014	-	-	-	-	-	-	-	-	-	-	0.833	0.240
163	Asp	m3	S^2,R_{ex}	-	0.810	0.044	-	-	-	-	-	- -	-	-	-	2.323	0.910	
164	Arg	m3	S^2,R_{ex}	-	0.793	0.033	-	-	-	-	-	-	-	-	-	-	1.965	0.690
165	Ile	m3	S^2,R_{ex}	-	0.784	0.026	-	-	-	-	-	-	-	-	-	-	4.868	0.590
**166**	**Glu**	**o.l.**	**-**	**-**	**-**	**-**	**-**	**-**	**-**	**-**	**-**	**-**	**-**	**-**	**-**	**-**	**-**	**-**
167	Pro	-	-	-	-	-	-	-	-	-	-	-	-	-	-	-	-	-
168	Asp	o.l.	-	-	-	-	-	-	-	-	-	-	-	-	-	-	-	-
169	Leu	o.l.	-	-	-	-	-	-	-	-	-	-	-	-	-	-	-	-
170	Asn	o.l.	-	-	-	-	-	-	-	-	-	-	-	-	-	-	-	-
171	Glu	m0	-	-	-	-	-	-	-	-	-	-	-	-	-	-	-	-
172	Gly	o.l.	-	-	-	-	-	-	-	-	-	-	-	-	-	-	-	-
173	Lys	n.o.	-	-	-	-	-	-	-	-	-	-	-	-	-	-	-	-
174	Leu	o.l.	-	-	-	-	-	-	-	-	-	-	-	-	-	-	-	-
175	Gly	o.l.	-	-	-	-	-	-	-	-	-	-	-	-	-	-	-	-
176	Asp	m1	S^2	-	0.893	0.007	-	-	-	-	-	-	-	-	-	-	-	-
177	Leu	m3	S^2,R_{ex}	-	0.894	0.019	-	-	-	-	-	-	-	-	-	-	1.419	0.370
178	Arg	m3	S^2,R_{ex}	-	0.943	0.013	-	-	-	-	-	-	-	-	-	-	1.331	0.230
179	Asp	m9	R_{ex}	-	-	-	-	-	-	-	-	-	-	-	-	-	1.963	0.740
180	Thr	m3	S^2,R_{ex}	yes	0.894	0.021	-	-	-	-	-	-	-	-	-	-	2.615	0.440
181	Thr	o.l.	-	-	-	-	-	-	-	-	-	-	-	-	-	-	-	-

Table S2: cTEM-17m model-free analysis results (continued)

#	aa	Model	Parameters	Diffusion	S^2	ΔS^2	S^2_f	ΔS^2_f	S^2_s	ΔS^2_s	τ_e	$\Delta\tau_e$	τ_f	$\Delta\tau_f$	τ_s	$\Delta\tau_s$	$^{600}R_{ex}$	$\Delta^{600}R_{ex}$
				core ?							ps	ps	ps	ps	ps	ps	s^{-1}	s^{-1}
182	Thr	o.l.	-	-	-	-	-	-	-	-	-	-	-	-	-	-	-	-
183	Pro	-	-	-	-	-	-	-	-	-	-	-	-	-	-	-	-	-
184	Lys	m1	S^2	yes	0.916	0.015	-	-	-	-	-	-	-	-	-	-	-	-
185	Ala	m3	S^2,R_{ex}	yes	0.913	0.014	-	-	-	-	-	-	-	-	-	-	0.778	0.250
186	Ile	m1	S^2	yes	0.933	0.009	-	-	-	-	-	-	-	-	-	-	-	-
187	Ala	o.l.	-	-	-	-	-	-	-	-	-	-	-	-	-	-	-	-
188	Ser	m1	S^2	yes	0.894	0.007	-	-	-	-	-	-	-	-	-	-	-	-
189	Thr	m3	S^2,R_{ex}	yes	0.893	0.012	-	-	-	-	-	-	-	-	-	-	1.091	0.230
190	Leu	m1	S^2	yes	0.965	0.010	-	-	-	-	-	-	-	-	-	-	-	-
191	Arg	m1	S^2	yes	0.924	0.010	-	-	-	-	-	-	-	-	-	-	-	-
192	Lys	m1	S^2	yes	0.935	0.008	-	-	-	-	-	-	-	-	-	-	-	-
193	Leu	m1	S^2	yes	0.906	0.009	-	-	-	-	-	-	-	-	-	-	-	-
194	Leu	o.l.	-	-	-	-	-	-	-	-	-	-	-	-	-	-	-	-
195	Thr	m1	S^2	yes	0.918	0.011	-	-	-	-	-	-	-	-	-	-	-	-
196	Gly	m5	S^2_f,S^2,τ_s	-	0.780	0.026	0.913	0.008	0.855	0.029	-	-	-	-	5025.258	2755.189	-	-
197	Glu	m2	S^2,τ_e	-	0.859	0.009	-	-	-	-	39.514	7.943	-	-	-	-	-	-
198	Leu	m5	S^2_f,S^2,τ_s	-	0.720	0.006	0.821	0.006	0.878	0.006	-	-	-	-	909.163	63.336	-	-
199	Leu	o.l.	-	-	-	-	-	-	-	-	-	-	-	-	-	-	-	-
200	Thr	m1	S^2	-	0.887	0.011	-	-	-	-	-	-	-	-	-	-	-	-
201	Leu	m5	S^2_f,S^2,τ_s	yes	0.870	0.013	0.910	0.006	0.956	0.012	-	-	-	-	7048.947	3308.117	-	-
202	Ala	m2	S^2,τ_e	yes	0.923	0.005	-	-	-	-	30.967	10.672	-	-	-	-	-	-
203	Ser	m1	S^2	yes	0.907	0.005	-	-	-	-	-	-	-	-	-	-	-	-
204	Arg	m3	S^2,R_{ex}	yes	0.929	0.011	-	-	-	-	-	-	-	-	-	-	0.464	0.190
205	Gln	m1	S^2	yes	0.951	0.011	-	-	-	-	-	-	-	-	-	-	-	-
206	Gln	m1	S^2	yes	0.921	0.007	-	-	-	-	-	-	-	-	-	-	-	-
207	Leu	m7	S^2_f,τ_s,S^2,R_{ex}	yes	0.095	0.054	0.432	0.032	0.221	0.108	-	-	-	-	2724.585	225.844	6.329	0.630
208	Ile	m3	S^2,R_{ex}	yes	0.915	0.021	-	-	-	-	-	-	-	-	-	-	1.398	0.400
209	Asp	m1	S^2	yes	0.959	0.011	-	-	-	-	-	-	-	-	-	-	-	-
210	Trp	m3	S^2,R_{ex}	yes	0.923	0.012	-	-	-	-	-	-	-	-	-	-	0.545	0.220
211	Met	m1	S^2	yes	0.966	0.014	-	-	-	-	-	-	-	-	-	-	-	-
212	Glu	o.l.	-	-	-	-	-	-	-	-	-	-	-	-	-	-	-	-
213	Ala	m9	R_{ex}	-	-	-	-	-	-	-	-	-	-	-	-	-	3.826	0.690
214	Asp	n.o.	-	-	-	-	-	-	-	-	-	-	-	-	-	-	-	-
215	Lys	o.l.	-	-	-	-	-	-	-	-	-	-	-	-	-	-	-	-
216	Val	n.o.	-	-	-	-	-	-	-	-	-	-	-	-	-	-	-	-
217	Ala	n.o.	-	-	-	-	-	-	-	-	-	-	-	-	-	-	-	-
218	Gly	n.o.	-	-	-	-	-	-	-	-	-	-	-	-	-	-	-	-
219	Pro	-	-	-	-	-	-	-	-	-	-	-	-	-	-	-	-	-
220	Leu	n.o.	-	-	-	-	-	-	-	-	-	-	-	-	-	-	-	-
221	Leu	n.o.	-	-	-	-	-	-	-	-	-	-	-	-	-	-	-	-
222	Arg	o.l.	-	-	-	-	-	-	-	-	-	-	-	-	-	-	-	-
223	Ser	o.l.	-	-	-	-	-	-	-	-	-	-	-	-	-	-	-	-
224	Ala	m1	S^2	-	0.889	0.011	-	-	-	-	-	-	-	-	-	-	-	-
225	Leu	m3	S^2,R_{ex}	-	0.829	0.022	-	-	-	-	-	-	-	-	-	-	1.270	0.440
226	Pro	-	-	-	-	-	-	-	-	-	-	-	-	-	-	-	-	-
227	Ala	m5	S^2_f,S^2,τ_s	-	0.819	0.010	0.858	0.006	0.955	0.009	-	-	-	-	3487.213	2408.675	-	-
228	Gly	m1	S^2	-	0.839	0.014	-	-	-	-	-	-	-	-	-	-	-	-
229	Trp	m3	S^2,R_{ex}	-	0.909	0.012	-	-	-	-	-	-	-	-	-	-	0.621	0.200
230	Phe	m3	S^2,R_{ex}	yes	0.898	0.020	-	-	-	-	-	-	-	-	-	-	2.100	0.380
231	Ile	m3	S^2,R_{ex}	yes	0.879	0.036	-	-	-	-	-	-	-	-	-	-	4.032	0.840
232	Ala	o.l.	-	-	-	-	-	-	-	-	-	-	-	-	-	-	-	-

Table S2: cTEM-17m model-free analysis results (continued)

#	aa	Model	Parameters	Diffusion core ?	S^2	ΔS^2	S_f^2	ΔS_f^2	S_s^2	ΔS_s^2	τ_e ps	$\Delta\tau_e$ ps	τ_f ps	$\Delta\tau_f$ ps	τ_s ps	$\Delta\tau_s$ ps	$^{600}R_{ex}$ s^{-1}	$\Delta^{600}R_{ex}$ s^{-1}
233	Asp	m0	-	yes	-	-	-	-	-	-	-	-	-	-	-	-	-	-
234	Lys	n.o.	-	-	-	-	-	-	-	-	-	-	-	-	-	-	-	-
235	Ser	n.o.	-	-	-	-	-	-	-	-	-	-	-	-	-	-	-	-
236	Gly	n.o.	-	-	-	-	-	-	-	-	-	-	-	-	-	-	-	-
237	Ala	n.o.	-	-	-	-	-	-	-	-	-	-	-	-	-	-	-	-
238	Gly	n.o.	-	-	-	-	-	-	-	-	-	-	-	-	-	-	-	-
240	Glu	n.o.	-	-	-	-	-	-	-	-	-	-	-	-	-	-	-	-
241	Arg	n.o.	-	-	-	-	-	-	-	-	-	-	-	-	-	-	-	-
242	Gly	n.o.	-	-	-	-	-	-	-	-	-	-	-	-	-	-	-	-
243	Ser	n.o.	-	-	-	-	-	-	-	-	-	-	-	-	-	-	-	-
244	Arg	n.o.	-	-	-	-	-	-	-	-	-	-	-	-	-	-	-	-
245	Gly	o.l.	-	-	-	-	-	-	-	-	-	-	-	-	-	-	-	-
246	Ile	o.l.	-	-	-	-	-	-	-	-	-	-	-	-	-	-	-	-
247	Ile	o.l.	-	-	-	-	-	-	-	-	-	-	-	-	-	-	-	-
248	Ala	m3	S^2,R_{ex}	yes	0.908	0.020	-	-	-	-	-	-	-	-	-	-	0.906	0.380
249	Ala	m3	S^2,R_{ex}	yes	0.911	0.022	-	-	-	-	-	-	-	-	-	-	1.308	0.420
250	Leu	m3	S^2,R_{ex}	yes	0.888	0.023	-	-	-	-	-	-	-	-	-	-	1.725	0.490
251	Gly	m3	S^2,R_{ex}	yes	0.887	0.019	-	-	-	-	-	-	-	-	-	-	1.435	0.380
252	Pro	-	-	-	-	-	-	-	-	-	-	-	-	-	-	-	-	-
254	Asp	m3	S^2,R_{ex}	-	0.908	0.018	-	-	-	-	-	-	-	-	-	-	1.905	0.410
255	Gly	m9	R_{ex}	-	-	-	-	-	-	-	-	-	-	-	-	-	4.814	0.560
256	Lys	m1	S^2	-	0.906	0.005	-	-	-	-	-	-	-	-	-	-	-	-
257	Pro	-	-	-	-	-	-	-	-	-	-	-	-	-	-	-	-	-
258	Ser	m0	-	-	-	-	-	-	-	-	-	-	-	-	-	-	-	-
259	Arg	o.l.	-	-	-	-	-	-	-	-	-	-	-	-	-	-	-	-
260	Ile	m1	S^2	yes	0.908	0.014	-	-	-	-	-	-	-	-	-	-	-	-
261	Val	m1	S^2	yes	0.915	0.019	-	-	-	-	-	-	-	-	-	-	-	-
262	Val	m1	S^2	yes	0.891	0.018	-	-	-	-	-	-	-	-	-	-	-	-
263	Ile	o.l.	-	-	-	-	-	-	-	-	-	-	-	-	-	-	-	-
264	Tyr	m9	R_{ex}	yes	-	-	-	-	-	-	-	-	-	-	-	-	1.591	0.560
265	Thr	o.l.	-	-	-	-	-	-	-	-	-	-	-	-	-	-	-	-
266	Thr	n.o.	-	-	-	-	-	-	-	-	-	-	-	-	-	-	-	-
267	Gly	n.o.	-	-	-	-	-	-	-	-	-	-	-	-	-	-	-	-
268	Ser	m0	-	-	-	-	-	-	-	-	-	-	-	-	-	-	-	-
269	Gln	o.l.	-	-	-	-	-	-	-	-	-	-	-	-	-	-	-	-
270	Ala	o.l.	-	-	-	-	-	-	-	-	-	-	-	-	-	-	-	-
271	Thr	m2	S^2,τ_e	-	0.925	0.015	-	-	-	-	69.313	60.329	-	-	-	-	-	-
272	Met	n.o.	-	-	-	-	-	-	-	-	-	-	-	-	-	-	-	-
273	Asp	m1	S^2	yes	0.950	0.010	-	-	-	-	-	-	-	-	-	-	-	-
274	Glu	m3	S^2,R_{ex}	yes	0.889	0.012	-	-	-	-	-	-	-	-	-	-	1.308	0.220
275	Arg	o.l.	-	-	-	-	-	-	-	-	-	-	-	-	-	-	-	-
276	Asn	o.l.	-	-	-	-	-	-	-	-	-	-	-	-	-	-	-	-
277	Arg	o.l.	-	-	-	-	-	-	-	-	-	-	-	-	-	-	-	-
278	Gln	m1	S^2	yes	0.940	0.024	-	-	-	-	-	-	-	-	-	-	-	-
279	Ile	o.l.	-	-	-	-	-	-	-	-	-	-	-	-	-	-	-	-
280	Ala	m2	S^2,τ_e	yes	0.853	0.008	-	-	-	-	67.061	9.800	-	-	-	-	-	-
281	Glu	o.l.	-	-	-	-	-	-	-	-	-	-	-	-	-	-	-	-
282	Ile	m3	S^2,R_{ex}	yes	0.906	0.023	-	-	-	-	-	-	-	-	-	-	1.478	0.470
283	Gly	m0	-	yes	-	-	-	-	-	-	-	-	-	-	-	-	-	-
284	Ala	m3	S^2,R_{ex}	yes	0.936	0.020	-	-	-	-	-	-	-	-	-	-	1.028	0.390
285	Ser	m1	S^2	yes	0.919	0.013	-	-	-	-	-	-	-	-	-	-	-	-
286	Leu	m1	S^2	yes	0.894	0.011	-	-	-	-	-	-	-	-	-	-	-	-

Table S2: cTEM-17m model-free analysis results (continued)

**#**	**aa**	**Model**	**Parameters**	**Diffusion core ?**	S^2	ΔS^2	S^2_f	ΔS^2_f	S^2_s	ΔS^2_s	τ_e	$\Delta\tau_e$	τ_f	$\Delta\tau_f$	τ_s	$\Delta\tau_s$	$^{600}R_{ex}$	$\Delta^{600}R_{ex}$
											**ps**	**ps**	**ps**	**ps**	**ps**	**ps**	$\mathbf{s^{-1}}$	$\mathbf{s^{-1}}$
287	Ile	m3	S^2,R_{ex}	yes	0.920	0.030	-	-	-	-	-	-	-	-	-	-	5.911	0.790
288	Lys	o.l.	-	-	-	-	-	-	-	-	-	-	-	-	-	-	-	-
289	His	m1	S^2	-	0.900	0.013	-	-	-	-	-	-	-	-	-	-	-	-
290	Trp	m1	S^2	-	0.853	0.009	-	-	-	-	-	-	-	-	-	-	-	-

- The diffusion tensor was minimised using residues in secondary structures (*i.e.* with 'Diffusion core → yes', *N*=100). Other residues (*N*=67) were excluded from diffusion tensor optimisation.
- R_{ex} parameters are calculated for a magnetic field of 600 MHz.
- Values presented here are rounded to three decimals. Exact values can be obtained from the BMRB (accession number 16598).
- Important active site residues (Ser^{70}, Lys^{73}, Tyr^{105}, Ser^{130}, Glu^{166}, and Arg^{234}) are shown in bold red while residues from the Ω loop (residues 161–179) are coloured blue.
- n-ter: N-terminus amine (not observable).
- n.o.: non-observed N-H resonances (not assigned).
- o.l.: overlapped N-H resonances.
