# Supplementary material for: Chimeric β-Lactamases: Global Conservation of Parental Function and Fast Time-Scale Dynamics with Increased Slow Motions
Source: PLoS One. 2012 Dec 21;7(12):e52283. doi: 10.1371/journal.pone.0052283 (PMC3528772; doi:10.1371/journal.pone.0052283)
Supplement: Supporting Information S1 — (DOC) [file pone.0052283.s010.doc]

**Supporting Information**

TABLE OF CONTENTS

I) β-Lactam substrates

Figure S1: β-Lactam substrates used in this work.

II) Representative kinetics data

Figure S2: Michaelis-Menten analysis of carbenicillin hydrolysis, monitored in 10 cm path-length cells.

III) Long-term stability of chimeric β-lactamase cTEM-17m

Figure S3: Specific activity of the chimera cTEM-17m at 0.8 mM monitored over a one-week period at 31.5 ˚C under the NMR sample conditions.

IV) Supplementary NMR data

Figure S4: 2D ^15^N-HSQC of [^15^N/^13^C]-labelled cTEM-17m.

Figure S5: cTEM-17m ^15^N spin relaxation data.

Figure S6: Consistency test results.

Figure S7: Representation of cTEM-17m diffusion tensor.

Table S1: cTEM-17m ^15^N spin relaxation data.

Table S2: cTEM-17m model-free parameters.

References

**III) Long-term stability of chimeric β-lactamase cTEM-17m**

Stability testing was done using a CENTA-based chromogenic activity assay to assess the overall stability of the chimera cTEM-17m under buffer and temperature conditions used to acquire the NMR data. Specifically, a lyophilized sample of labelled cTEM-17m was solubilized (in 3mM imidazole, 0.1% azide at pH=6.8) to a final concentration of approximately 0.8mM and kept at 31.5˚C (the temperature used for NMR experiments). The specific activity was monitored over a one-week period (**Fig**. **S3**).

Figure S3 illustrates that the cTEM-17m sample lost less than 20% specific activity over a one-week period under the NMR sample conditions. The 0.8 mM protein concentration tested was rigorous, as the NMR backbone assignment and relaxation study were performed at only 0.4mM, where less aggregation would be expected. The presence or absence of low NaCl concentrations (*i.e*.10 mM, data not shown) had no significant influence on the activity during the course of this assay. This confirms the fitness of cTEM-17m for the required week-long NMR data acquisition.

References:

Bebrone, C., C. Moali, et al. (2001). "CENTA as a chromogenic substrate for studying beta-lactamases." Antimicrobial Agents and Chemotherapy **45**(6): 1868-1871.

Morin, S., C. M. Clouthier, et al. (2010). "Backbone resonance assignments of an artificially engineered TEM-1/PSE-4 Class A beta-lactamase chimera." Biomolecular NMR assignments **4**(2): 127-130.
